# Numerical backup protection scheme based on alienation indices of voltage and current measurements practically applied to synchronous generators

**DOI:** 10.1038/s41598-026-51239-x

**Published:** 2026-05-18

**Authors:** R. A. Mahmoud, Mohamed Adel Esmaeel Salama

**Affiliations:** 1 Department of Electrical Power and Machines Engineering (PME), College of Engineering Science & Technology, Misr University for Science and Technology (MUST), 6th of October City, Giza, Egypt; 2 Department of Electrical Power and Machines Engineering, Faculty of Helwan Engineering, Capital University (formerly Helwan University), Cairo, Egypt

**Keywords:** Synchronous machine, Fault detection, Primary protection, Secondary protection, Alienation criteria, Series and shunt faults, Unbalance estimation, Energy science and technology, Engineering, Mathematics and computing

## Abstract

Differential current relays are the primary protection for detecting fault disturbances within the protection zone of three-phase synchronous generator stator windings. This paper presents a numerical backup protection scheme, based on the alienation algorithm, which serves as a redundant protection when the main differential current relay fails to isolate stator winding faults. The proposed method quantifies a set of alienation indices derived from fifteen Pearson correlation coefficients, calculated for the three-phase voltages and currents measured at the load terminals of the generator stator windings. A novel set of tripping characteristic curves, formulated from the alienation indices, enables the implementation of secondary protection functions. The method effectively detects fault disturbances and voltage/current imbalance conditions in the three-phase generator signals. The experimental validation is performed using a motor–generator set equipped with voltage and current transformers installed at the generator load terminals. The system hardware is emulated through a DAC and personal computer, while the alienation algorithm is implemented and processed using the LABVIEW software environment. Extensive experimental investigations were carried out to validate the proposed method under various fault scenarios affecting the generator. The results demonstrate the alienation algorithm’s notable simplicity, effectiveness, reliability, and efficiency. Quantitatively, the protection scheme achieved security and dependability levels exceeding 99.80%, while its reliability and accuracy surpassed 99.60%. Moreover, the proposed scheme reduces the protection malfunction rate to only 0.37%. Compared to the coherence-based alienation method for analyzing voltage and current signals, the correlation-based alienation method provides superior response speed, improved performance metrics, greater accuracy, and a more reliable assessment of asymmetry and disturbance levels. An alienation tripping-time curve is created for the backup protection scheme, enabling time-delayed tripping under fault conditions, and its operating speed can be flexibly adjusted to meet specific protection requirements.

**Supplementary Information:**

The online version contains supplementary material available at 10.1038/s41598-026-51239-x.

## Introduction

### Motivation and incitement

For AC machine protection, protective relays are generally classified into primary and secondary (backup) relays. Phase differential current relays provide the primary protection for the stator windings of AC machines^1^. Secondary protection safeguards the machine against external fault disturbances and compensates for any malfunction of the primary protection, thereby providing a redundant protective layer^2^. Backup protection for AC machines can be applied to various components, including the machine stator winding, field winding, speed governor, automatic voltage regulator (AVR), and circuit breaker. For example, overvoltage, over-fluxing, and loss-of-excitation relays serve as backup protection for the AVR, while underfrequency and overfrequency relays act as governor backups. The stator windings can be protected using phase overcurrent, earth fault, restricted earth fault, voltage-controlled time overcurrent, voltage/current imbalance, low forward/reverse power, and distance relays^3^. The selection of these protections depends on the machine’s capacity and its connection to the power network^4^. Implementing backup protections requires voltage and/or current transformers installed at the neutral and/or load terminals of the machine^5^. This paper introduces a numerical backup protection scheme for synchronous generators based on alienation indices derived from voltage and current measurements. The proposed method is designed to detect and diagnose a wide range of fault disturbances, thereby providing an additional protective layer to enhance generator lifespan, efficiency, and reliability^6^.

### Literature review

Numerous studies have investigated traditional backup protection schemes against overcurrent in electrical networks. Some have compared conventional methods with adaptive systems that automatically adjust tripping thresholds based on backup system loading and the direction of the tripping current. Optimization algorithms have also been applied to determine optimal settings for overcurrent relays assigned to specific fault zones^7^. In another work, generator and transformer power curves were used in combination to locate short circuits based on current measurements and the power curve. The results confirmed the algorithms’ rapid capability to determine whether the fault occurred inside or outside the generator and transformer under various loading conditions, including no-load operation^8^. Other research has introduced the Deep Convolutional Transfer Learning Network (DCTLN) approach, which models the machine’s condition under normal operation. Although obtaining complete baseline data is challenging, this method has achieved high accuracy in differentiating short circuits from overloads. However, it focuses solely on machine-level faults and requires comprehensive normal-condition datasets for each network component to ensure effective isolation under abnormal conditions^9^. Fuzzy control techniques have also been used to reset differential relays in short-circuit events where conventional overcurrent protection fails. These approaches have relied on high-precision measurement devices and have been limited to protecting generators against internal short circuits^10,11^. Another study implemented a Transient Frequency Component (TFC) scheme to assess generator coherence by analyzing speed deviation signals and voltage angle differences, with performance compared across various algorithms^12,13^.

Some researchers have highlighted that AI-based fault diagnosis devices for synchronous generators often exhibit low accuracy due to long computation times and excessive processing requirements^14^. The accuracy of maximum inter-cycle error classification on a small-scale synchronous generator model has been improved using the Nuisance Attribute Projection (NAP) algorithm^15^. Significant efforts have also been devoted to distinguishing between internal and external faults in synchronous generators to improve protection relay operating times^16–21^. For induction machines, stator current analysis combined with the Park transform has been used to classify stator winding faults. A self-organizing neural network served as the classifier, comparing stator current signals in faulted and healthy conditions under varying load levels. Experimental findings demonstrated that the method could reliably distinguish between normal and faulted states, accurately identify the affected phase, and determine the severity of the fault^22,23^.

In^24^, a magnetic flux-based algorithm was proposed to detect reverse power conditions in synchronous generators. Research in^25^ introduced an integrated protection algorithm that employs a specially designed indicator to detect faults across multiple network layers, including transmission, distribution, and microgrid systems. The study in^26^ investigated diode rectifier faults in brushless synchronous generators using the discrete wavelet transform (DWT). Similarly^27^, presented an Adaptive Neuro-Fuzzy Inference System (ANFIS) model for diagnosing inter-turn short-circuit (ITSC) and broken rotor bar (BRB) faults in squirrel cage induction motors (SC-IMs), also utilizing DWT. Thus, both^26^ and^27^ demonstrated the effectiveness of DWT in diagnosing faults in brushless synchronous generators and SC-IMs. In^28^, an extended Kalman filter (EKF)-based model was proposed to detect inter-turn short-circuit faults in switched reluctance motors (SRMs). Furthermore, the same paper introduced a novel application of waveform transformers for stator fault diagnosis and protection in synchronous generators, relying on instantaneous powers of waveform transformer coefficients derived from line voltages and currents. A comprehensive review in^29^ examined various machine learning and artificial intelligence–based diagnostic methods for detecting incipient bearing faults, such as pinholes and surface scratches, in induction motors.

In this paper, a numerical backup protection scheme for synchronous generators is proposed to operate when the primary high-speed differential overcurrent protection fails to detect or clear an internal fault. The method detects and evaluates the severity of fault disturbances in the generator’s voltage and current waveforms using alienation coefficients^30,31^, which are derived from Pearson correlation-based calculations^32^. The alienation settings of the proposed technique define the tripping criteria for synchronous generator protection, with the scheme functioning as a voltage-restrained time overcurrent element to safeguard the three-phase stator windings. Upon detecting a fault and confirming that the primary protection has not operated, the scheme issues a trip command to the appropriate generator circuit breaker(s). Proper coordination and an intentional time delay are incorporated to ensure activation only in cases of phase or earth faults where the primary protection is ineffective.

For clarification, there are distinct differences between the currently submitted paper and the previously published article^31^. The present work employs a correlation-based alienation method, whereas the earlier study relied on a coherence-based alienation method for analyzing the voltage and current signals at the synchronous generator load terminals. Building upon the findings of^31^, this study demonstrates that the correlation-based approach achieves a faster response speed compared to the coherence-based algorithm. In addition, the proposed protection scheme exhibits superior performance in terms of accuracy, security, dependability, and reliability, even though the same power system setup has been subjected to more extensive testing scenarios. As a result, the proposed scheme limits the protection malfunction rate to a negligible level. A further contribution of this work is the formulation of a novel alienation-based tripping-time curve for the backup protection scheme, which ensures coordinated, time-delayed tripping under fault conditions.

Furthermore, recent Artificial Intelligence (AI)–based studies^33^, including machine learning techniques^34–36^, deep learning models^37^, and hybrid data-driven approaches^38^, have been increasingly employed to continuously monitor electrical machines and power grids, predict faults and outages before they occur, and optimize overall energy efficiency. Despite these advantages, AI-based protection schemes exhibit several limitations, such as the need for large and diverse training datasets, limited generalization capability under unseen or rare fault conditions, and vulnerability to misoperation during temporary disturbances, noise, or measurement distortions. These shortcomings further reinforce the motivation for developing the proposed alienation-coefficient–based backup protection method.

### Paper contributions

The main contributions of this work can be summarized as follows:

1. Alienation-based backup protection – Alienation indices, derived from Pearson correlation coefficients of voltage and current measurements, are introduced to develop a novel digital backup protection scheme for the three-phase stator windings of synchronous machines. This approach can also be extended to protect the three-phase stator windings of other AC machines and power transformers.
2. New operating characteristic curves – The proposed approach enables the development of new quadratic tripping characteristics based on mutual- and serial-alienation indices, facilitating the discrimination between fault and no-fault conditions, the distinction of balanced and unbalanced states, and the assessment of voltage and current asymmetry severity.
3. Auto-alienation-based protection tripping time curve – An auto-alienation-based protection tripping time curve is proposed for the backup protection scheme, enabling time-delayed tripping under fault conditions.
4. Mathematical model for unbalance assessment – A new mathematical model employing alienation coefficients is formulated to evaluate voltage and current unbalance in AC equipment. This model is applicable to a variety of electrical machines for accurate imbalance assessment.
5. Controllable protection performance – The alienation-based method allows for adjustable protection sensitivity, security, dependability, and operating speed, enabling adaptation to different protection requirements.

### Paper organization

This manuscript is structured as follows: Sect.  2 presents the fundamentals of the alienation-based protection scheme, detailing the algorithmic procedure and the tripping characteristic curves of the proposed method. Section  3 describes the experimental setup and the specifications of its power components, which are used to evaluate the effectiveness and accuracy of the developed algorithm under practical operating conditions. Section  4 reports and discusses the experimental findings. Section  5 outlines the advantages of the proposed backup protection scheme and provides a comprehensive comparison with other recently published approaches. Finally, Sect.  6 summarizes the key quantitative results and offers concluding remarks.

## Alienation-based protection scheme

### Justification

During a fault in a synchronous generator or its connected power network, several parameters may change, including:

1. RMS values of terminal voltages decrease,
2. RMS values of terminal currents increase under fault or overload conditions,
3. Disturbances in frequency and phase angle of the electrical signals,
4. Variations in waveform shape, and.
5. Imbalance among the three-phase voltage and/or current signals.

To detect and respond to these changes, a numerical method based on the alienation algorithm is proposed, complemented by novel quadratic tripping characteristics for synchronous generator protection. This method requires real-time measurements of the three-phase voltage and current signals at the terminals of the protected system component. The numerical method based on the alienation algorithm operates as a backup protection system, providing redundancy in case the main differential current relay fails to isolate stator winding faults.

### Alienation coefficients

The determination coefficient is defined as the square of the correlation coefficient. Subtracting this value from unity yields the alienation coefficient (also called the non-determination coefficient), which represents the proportion of variance not shared between two variables. In essence, it measures the degree of non-association between variables and is highly sensitive to outliers. Consequently, alienation values may vary for imbalanced data when sampling errors are present.

The alienation coefficient is dimensionless and can be used to compare results across different studies involving different variables. Its magnitude ranges from 0.0 to + 1.0. A value of + 1.0 indicates that the variables lie exactly on a straight line, whereas lower values indicate weaker or nonlinear relationships.

In the proposed method, two types of alienation coefficients are used:

1. Serial-alienation coefficient – obtained from the serial-correlation coefficient, calculated for two datasets of the same electrical signal in one phase.
2. Mutual-alienation coefficient – derived from the mutual-correlation coefficient, calculated for either two different electrical signals from two different phases or for voltage and current signals of the same phase.

Here, the serial-alienation monitors the condition of each phase’s voltage or current signal individually, while mutual-alienation evaluates the relationships between phases or between voltage and current of the same phase. This enables the protection scheme to distinguish between acceptable and unacceptable imbalance and to differentiate normal operating conditions from abnormal events such as overloads, series and shunt faults, or CT saturation, using the criteria in Table 1.

#### Method for calculating alienation coefficient

The alienation coefficient (*A*_*fg*_) between the two datasets (*f(n)* and *g(n)*), each containing *N*_*w*_ samples of the electrical signal (*S*), is estimated using the following expression based on the determination coefficient (*D*_*fg*_)^30,31^:

1 $${A_{fg}}=1 - {D_{fg}}$$

It can also be expressed using the following equation in terms of the correlation coefficient (*r*_*fg*_)^32^:

2 $${A_{fg}}=1 - {({r_{fg}})^2}$$

In the proposed algorithm, the sample alienation is calculated using the following mathematical expression^30^:

3 $${A_{fg}}=1 - {(\frac{{{N_w}\sum\limits_{{n=1}}^{{n={N_w}}} {f(n)g(n)} - \sum\limits_{{n=1}}^{{n={N_w}}} {f(n)} \sum\limits_{{n=1}}^{{n={N_w}}} {g(n)} }}{{\sqrt {{N_w}\sum\limits_{{n=1}}^{{n={N_w}}} {(f(n)} {)^2} - {{(\sum\limits_{{n=1}}^{{n={N_w}}} {f(n)} )}^2}} \times \sqrt {{N_w}\sum\limits_{{n=1}}^{{n={N_w}}} {{{(g(n))}^2} - (\sum\limits_{{n=1}}^{{n={N_w}}} {g(n){)^2}} } } }})^2}$$

The statistical equation presented above provides a practical algorithm for evaluating sample alienation, where the computational efficiency primarily depends on the dataset size.

Where,

**Table:** 

*r*_fg_	The Pearson correlation coefficient computed between two corresponding datasets of the electrical signals (f(*n*) and g(*n*))
*A*_*fg*_	The alienation coefficient computed between two corresponding datasets of the electrical signals (*f(n)* and *g(n)*)
*f(n)*	The first dataset of the electrical signal *f(n)* extracted for correlation analysis
*g(n)*	The second dataset of the electrical signal *g(n)* extracted for correlation analysis
*n*	The sample point index,
*Fc*	The fundamental frequency of the AC signals, which is 50 Hz in this study
*Fs*	The sampling frequency rate from the Data Acquisition Card (DAC), set to 2.5 kHz in this study
*N*_*w*_	The sample size (dataset length) used in the algorithm, with the condition *N*_*w*_ *≤ N*_*s*_
*N*_*s*_	The number of samples per cycle for the measured electrical signal, calculated as *N*_*s*_*= F*_*s*_*/F*_*c*_*=* 50 samples/cycle, where *F*_*s*_ is the sampling frequency and *F*_*c*_​ is the signal frequency

Although Eq. (3) involves division and square-root operations, these are performed over a finite discrete-time data window, enabling efficient implementation in digital platforms. The algorithm requires only basic time-domain operations (addition, multiplication, division, and comparison) on voltage and current samples, with normalization applied to prevent numerical overflow. Unlike conventional RMS- or Fourier-based methods, it does not rely on trigonometric functions or spectral analysis, resulting in a lower computational burden that is well suited for DSP-based relay implementation.

### Tripping logic

In the proposed strategy, the electrical signals measured at the synchronous generator load terminals using three-phase voltage and current transformers are named as follows: *v*_*a*_*(n)*,* v*_*b*_*(n)*, *v*_*c*_*(n)*, *i*_*a*_*(n)*,* i*_*b*_*(n)* and *i*_*c*_*(n)*, respectively. The nine mutual-alienation coefficients are denoted as *Av*_*ab*_, *Av*_*bc*_, *Av*_*ca.*_, *Ai*_*ab*_, *Ai*_*bc*_, *Ai*_*ca.*_, *Avi*_*a*_, *Avi*_*b*_ and *Avi*_*c*_, while the six serial-alienation coefficients are designated as *Av*_*a*_, *Av*_*b*_, *Av*_*c*_, *Ai*_*a*_, *Ai*_*b*_, and *Ai*_*c*_, These coefficients are utilized in the proposed protection method to determine the tripping decision for the protected synchronous generator.

#### Alienation threshold values

In this work, the threshold value *ΔJ* is assigned to the three mutual-alienation coefficients (*Av*_*ab*_, *Av*_*bc*_, and *Av*_*ca.*_), *Δk* to (*Ai*_*ab*_, *Ai*_*bc*_, and *Ai*_*ca.*_), and *Δz* to (*Avi*_*a*_, *Avi*_*b*_ and *Avi*_*c*_),

Similarly, the threshold value *ΔM* is assigned to the three serial-alienation coefficients (*Av*_*a*_, *Av*_*b*_, and *Av*_*c*_), while *ΔN* is assigned to (*Ai*_*a*_, *Ai*_*b*_, and *Ai*_*c*_).

The five threshold values (*ΔJ*,* Δk*,* Δz*,* ΔM*, and *ΔN*) are initially determined through extensive experimental testing and simulation under the specific operating conditions of the studied synchronous generator. While this empirical tuning ensures reliable performance for the investigated system, it may not be directly generalizable to generators with different ratings or parameters.

Varying each threshold affects protection sensitivity, fault detection accuracy, and false-alarm rates. The proposed scheme provides effective detection performance over reasonable variations of the threshold values. Appropriate threshold selection ensures that the scheme remains robust and not overly sensitive to minor variations.

Additionally, adaptation of the thresholds to generators with different capacities and impedances can be achieved by considering the expected variations in both mutual- and serial-alienation coefficients under nominal operating conditions. This approach facilitates the application of the proposed method to different generator configurations while maintaining reliable performance.

#### Tripping criteria

Table 1 presents the tripping criteria established for the proposed alienation-based digital protection scheme for synchronous generators. The table summarizes the five protection modules, including their respective input signals, estimated alienation coefficients, and the corresponding decision rules governing the tripping action.

**Table 1:** Tripping criteria defined for the proposed alienation-based digital protection scheme applied to synchronous generators.

Protection module	The input signals	Estimated alienation coefficient	Alienation coefficients domain	Machine status (balance/imbalance or normal/abnormal condition)	The protection module response (active/inactive)
	The selected alienation deviations (*ΔM*, *ΔN*, *ΔJ*, *Δk* and *Δz*) are 0.1, 0.1, 0.1, 0.1 and 0.75, respectively				
Module (1) to estimate the voltage imbalance level using the mutual-alienation coefficient between each two voltage signals	*v*_*a*_ *(n)* and *v*_*b*_ *(n)*	*Av*_*ab*_	*0.75 + ΔJ ≥ Av*_*ab*_ *≥ 0.75-ΔJ*, *0.75 + ΔJ ≥ Av*_*bc*_ *≥ 0.75-ΔJ* and *0.75 + ΔJ ≥ Av*_*ca.*_ *≥ 0.75-ΔJ*	**Normal and balanced voltages**	**Inactive**
	*v*_*b*_ *(n)* and *v*_*c*_ *(n)*	*Av*_*bc*_			
			*0.75 + ΔJ < Av*_*ab*_ *<0.75-ΔJ*, *0.75 + ΔJ <Av*_*bc*_ *<0.75-ΔJ* or *0.75 + ΔJ <Av*_*ca.*_ *<0.75-ΔJ*	**Voltages imbalance**	**Active**
	*v*_*c*_ *(n)* and *v*_*a*_ *(n)*	*Av*_*ca.*_			
Module (2) to measure the current imbalance level using the mutual-alienation coefficient between each two current signals	*i*_*a*_ *(n)* and *i*_*b*_ *(n)*	*Ai*_*ab*_	*0.75 + Δk ≥ Ai*_*ab*_ *≥ 0.75-Δk*, *0.75 + Δk ≥ Ai*_*bc*_ *≥ 0.75-Δk* and *0.75 + Δk ≥ Ai*_*ca.*_ *≥ 0.75-Δk*	**Normal and balanced currents**	**Inactive**
	*i*_*b*_ *(n)* and *i*_*c*_ *(n)*	*Ai*_*bc*_			
			*0.75 + Δk <Ai*_*ab*_ *<0.75-Δk*, *0.75 + Δk <Ai*_*bc*_ *<0.75-Δk* or *0.75 + Δk <Ai*_*ca.*_ *<0.75-Δk*	**Currents imbalance**	**Active**
	*i*_*c*_ *(n)* and *i*_*a*_ *(n)*	*Ai*_*ca.*_			
Module (3) to evaluate the power factor disturbance using the mutual-alienation coefficients between each phase voltage and current signals	*v*_*a*_ *(n)* and *i*_*a*_ *(n)*	*Avi*_*a*_	*0 ≤ Avi*_*a*_ *≤ ΔZ*, *0 ≤ Avi*_*b*_ *≤ ΔZ* and *0 ≤ Avi*_*c*_ *≤ ΔZ*	**Normal and balanced power factor**	**Inactive**
	*v*_*b*_ *(n)* and *i*_*b*_ *(n)*	*Avi*_*b*_			
			*ΔZ <Avi*_*a*_ *≤ 1*, *ΔZ <Avi*_*b*_ *≤ 1* or *ΔZ <Avi*_*c*_ *≤ 1*	**abnormal and unbalanced power factor**	**Active**
	*v*_*c*_ *(n)* and *i*_*c*_ *(n)*	*Avi*_*c*_			
Module (4) to specify the fault disturbance in the phase voltage using the serial-alienation coefficient for each phase voltage signal	*v*_*a*_ *(n)*	*Av*_*a*_	*0 ≤ Av*_*a*_ *≤ ΔM*, *0 ≤ Av*_*b*_ *≤ ΔM* and *0 ≤ Av*_*c*_ *≤ ΔM*	**Normal voltages**	**Inactive**
	*v*_*b*_ *(n)*	*Av*_*b*_			
			*ΔM <Av*_*a*_ *≤ 1*, *ΔM <Av*_*b*_ *≤ 1* or *ΔM <Av*_*c*_ *≤ 1*	**Abnormal voltages**	**Active**
	*v*_*c*_ *(n)*	*Av*_*c*_			
Module (5) to identify the fault disturbance in the phase current using the serial-alienation coefficient for each phase current signal	*i*_*a*_ *(n)*	*Ai*_*a*_	*0 ≤ Ai*_*a*_ *≤ ΔN*, *0 ≤ Ai*_*b*_ *≤ ΔN* and *0 ≤ Ai*_*c*_ *≤ ΔN*	**Normal currents**	**Inactive**
	*i*_*b*_ *(n)*	*Ai*_*b*_			
			*ΔN <Ai*_*a*_ *≤ 1*, *ΔN<Ai*_*b*_ *≤ 1* or *ΔN <Ai*_*c*_ *≤ 1*	**Abnormal currents**	**Active**
	*i*_*c*_ *(n)*	*Ai*_*c*_			

Significant values are in [bold and italics].

### Tripping curves

#### Alienation-based operating characteristics

In the proposed method, the protective relay operates when the calculated alienation values fall within the tripping zones of the developed closed-characteristic curves. Each alienation-based characteristic is configured through pre-defined settings specific to its intended operation. When the conditions outlined in Table 1 are satisfied, the relay issues a trip command to open the associated circuit breaker(s), thereby isolating the protected equipment from the rest of the system.

**Fig. 1:**
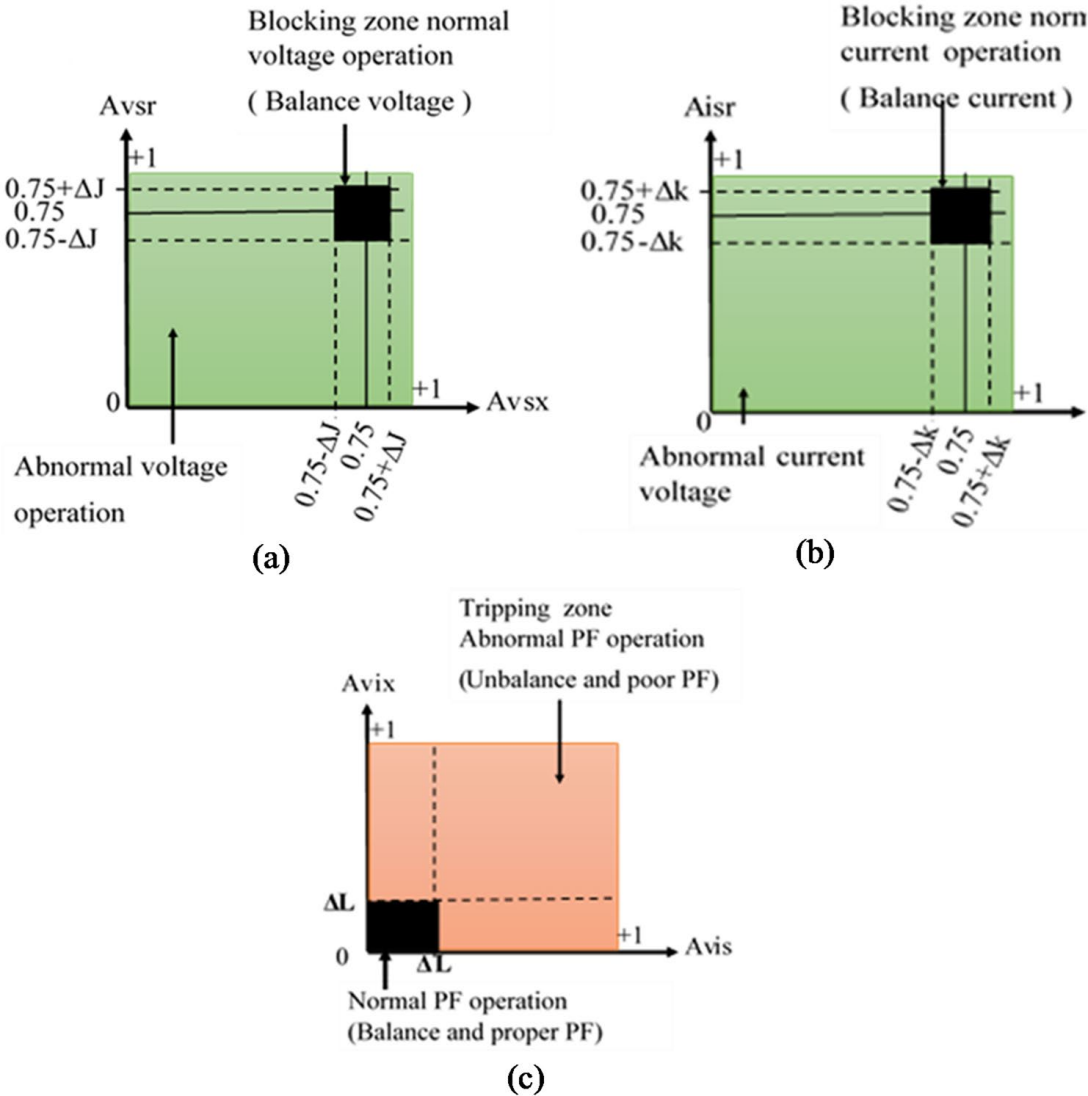
Tripping curve based on mutual-alienation for (a) Voltage imbalance, (b) current imbalance, and (c) power factor disturbance.

**Fig. 2:**
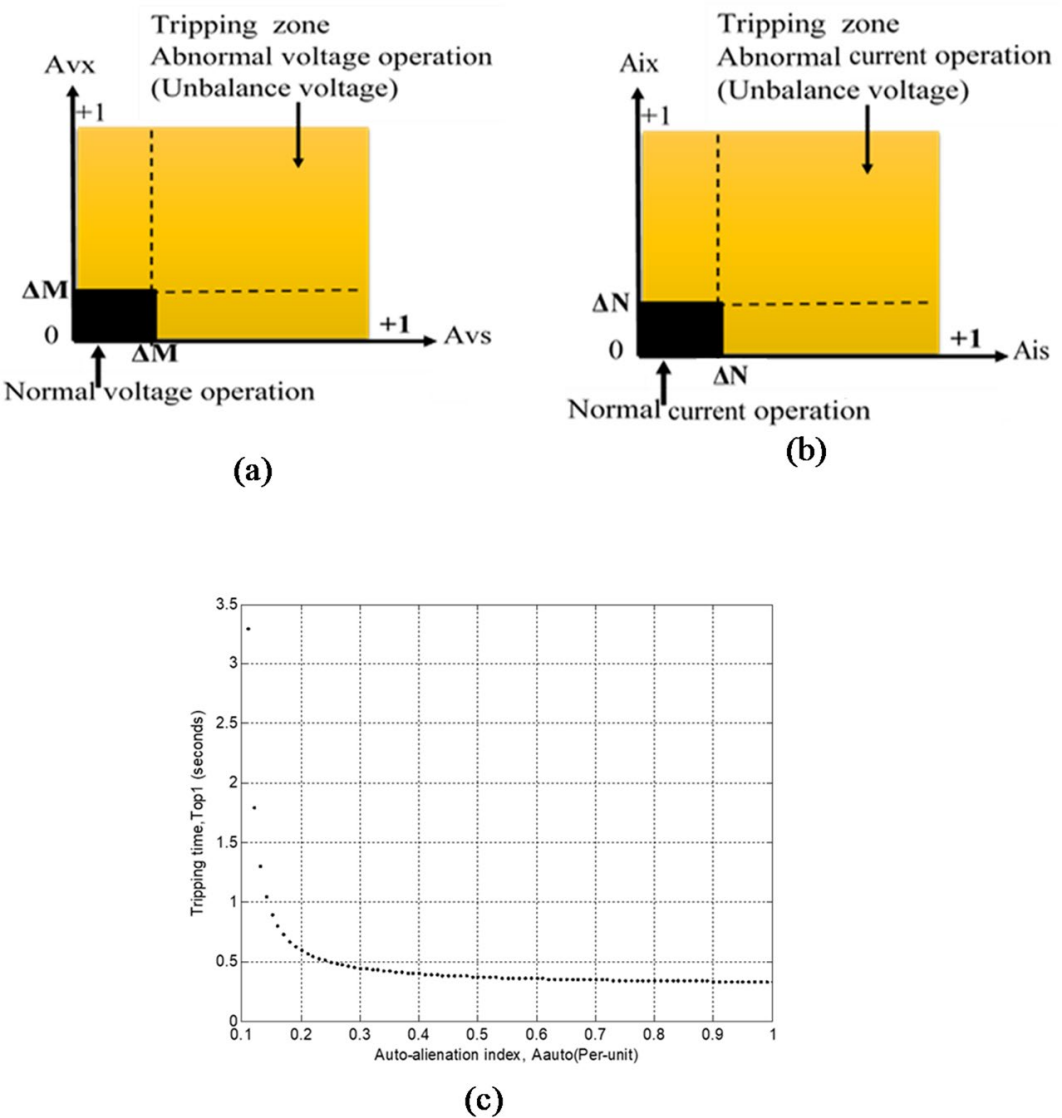

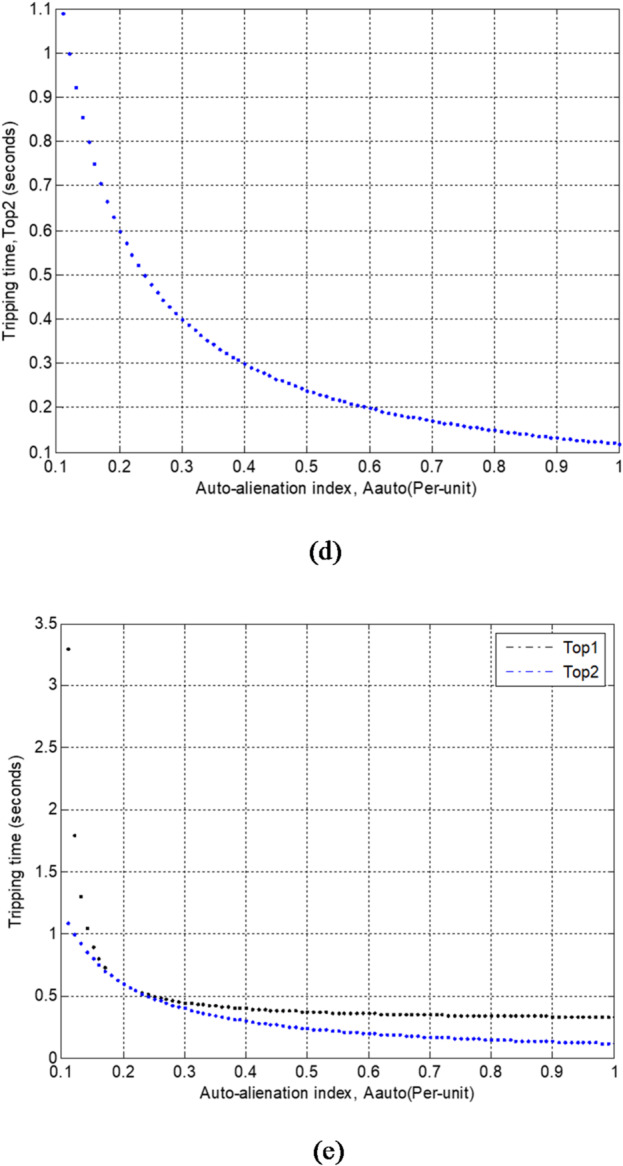
Tripping curve based on serial-alienation for (a) Voltage disturbance, and (b) Current disturbance. (c) A_Auto_-based T_op1_ tripping-time curve. (d) A_Auto_-based T_op2_ tripping-time curve. (e) A_Auto_-based T_op1_ and T_op2_ tripping-time curves.

The generator differential relay, serving as the main protection, is designed to clear only internal faults within the protected zone and must operate without intentional time delay to ensure immediate isolation. The proposed alienation-based backup protection is activated only when the primary protection fails to disconnect the faulted generator from the power system. Its operating time is controlled by the number of datasets pre-set in the protection algorithm. Figures 1(a–c) and 2(a–b) illustrate five categories of closed-tripping characteristics used to detect disturbances in voltage and current waveforms for the presented algorithm. Each operating characteristic is defined by alienation coefficient thresholds, whose values range between 0 and + 1. Furthermore, every quadratic characteristic includes two distinct regions:

1. Restraining region – blocks relay operation during normal, balanced, or acceptable imbalance conditions.
2. Tripping region – permits relay operation to isolate the faulty element during abnormal or unacceptable imbalance conditions.

The relay closed-characteristics shown in Figs. 1(a–c) and  2(a–b) are non-directional, meaning that they respond to faults occurring both in front of and behind the relay point. Consequently, the proposed protection system exhibits the following properties:

1. Non-directional operation – The relay detects faults on both sides of the relaying point, requiring an additional directional element for correct fault discrimination.
2. Non-uniform fault resistance coverage – The relay’s response varies depending on the fault resistance.
3. Sensitivity to system conditions – It may be affected by out-of-step conditions, power swings, or heavy loading of large generators due to the extensive area covered by the alienation quadrilateral tripping characteristics.
4. Adaptive response to arcing faults – The non-linear relationship between arc voltage and current can be captured using the alienation method. Under arcing or earth faults with additional resistance, the resistive component of the fault impedance increases, altering the impedance angle. The relay handles this by adapting the dataset size.
5. Directional discrimination – To prevent operation for faults outside the protected generator, a separate directional control element can be incorporated.
6. Deployment under severe disturbances – The proposed tripping protection can be applied during extreme power swings or out-of-step conditions, where system recovery is unlikely. In such cases, the scheme can strategically split the power system at a preferred location, a scenario often undetectable by conventional protection.

#### Alienation tripping-time curves

In this work, two tripping-time characteristics are proposed based on the running auto-alienation index (*A*_*auto*_*)*, the alienation pickup value *(A*_*pu*_*)*, and the time multiplier constants (*K*_*m*_ and *K*_*n*_). These characteristics are represented by two distinct mathematical formulas, which are described in detail in the following subsections:

1. The first type of tripping-time curve, formulated based on (*A*_*auto*_, *A*_*pu*_, and *K*_*m*_*)*, is applied as backup protection.
2. The second type of tripping-time curve, formulated based on (*A*_*auto*_, *A*_*pu*_, and *K*_*n*_*)*, is used for comparative analysis.

##### *A*_*Auto*_-based first tripping-time curve

It is well established that the differential overcurrent relay, serving as the primary protection, operates instantaneously, whereas the alienation-based protection functions as a backup scheme with a time-delayed tripping action. Accordingly, a new mathematical model is proposed to estimate the tripping time under fault conditions. This expression incorporates the actual auto-alienation estimator (*A*_*auto*_*)*, the alienation pickup value *(A*_*pu*_*)*, and the time multiplier (*K*_*m*_). Accordingly, Eq. (4) is employed to calculate the operating time (*T*_*op1*_).

4 $${T_{op1}}=\frac{{1/{K_m}}}{{1 - \left( {\frac{{{A_{pu}}}}{{{A_{Auto}}}}} \right)}},\;For\,{A_{Auto}}>{A_{pu}}$$

Where,

T_op1_: The operating time (in seconds) estimated using the auto-alienation (*A*_*auto*_), K_m_: The selected time multiplier (it is set to *K*_*m*_ *= 10/3*), A_pu_: The auto-alienation pickup value (it is set to *A*_*pu*_ *= 0.1* per-unit), and A_Auto_: The calculated auto-alienation index expressed in per-unit; it is a scalar and dimensionless quantity.

Equation (4) is invoked under the condition that a sudden change in any running auto-alienation index exceeds the predefined deviation threshold.

Figure 2(c) illustrates the proposed inverse alienation-time curve for estimating the fault tripping time (*T*_*op1*_), considering the settings *K*_*m*_ = 10/3 and *A*_*pu*_ = 0.10 per-unit. The selected values of *K*_*m*_ and *A*_*pu*_ are determined according to the prevailing system operating conditions.

##### *A*_*Auto*_-based second tripping-time curve

The relay uses the inverse law based on the calculated auto-alienation index (*A*_*Auto*_) to estimate the tripping time as follows:

5 $${T_{op2}}=\frac{{{K_n}}}{{\left( {\frac{{{A_{Auto}}}}{{{A_{pu}}}}} \right)}},\;\;For\;\;{A_{Auto}}>{A_{pu}}$$

Where,

T_op2_: ​ Relay tripping time (in seconds), K_n_: Time constant defining the speed of operation, *A*_*pu*_​: The auto-alienation pickup value (it is set to *A*_*pu*_ *= 0.1* per-unit), and A_Auto_: The calculated auto-alienation index expressed in per-unit; it is a scalar and dimensionless quantity.

This formulation ensures faster tripping for severe faults (large *A*_*Auto*_) while allowing time-delayed operation for mild disturbances, thus providing coordination with other protection devices. In other words, the inverse-time tripping characteristic illustrates that the relay operates progressively faster as the normalized indicator (*A*_*Auto*_*/A*_*pu*_) increases, thereby enhancing relay sensitivity. This characteristic ensures a balance between speed and coordination:

- For severe faults (i.e., large *A*_*Auto*_ */A*_*pu*_), the relay responds almost instantaneously, minimizing fault duration.
- For mild disturbances, the relay introduces a time delay, allowing upstream or downstream protection devices to operate first if necessary.

Such behavior improves overall selectivity, while maintaining high sensitivity to critical fault conditions.

Figure 2(a) exhibits the inverse relation between the conservative relay indicator measurement (*A*_*Auto*_) and the operating time (*T*_*op2*_). The curve is constructed using the inverse *A*_*Auto*_–time characteristic with settings *K*_*n*_ = 12/10 and *A*_*pu*_ = 0.10 per-unit. As *A*_*Auto*_ increases beyond the pickup threshold, the tripping time decreases, ensuring fast relay operation under severe disturbances and slower (or no) action during mild deviations.

Equations (4) and (5) are applied only when the running index (*A*_*Auto*_) exceed the predefined threshold (*A*_*pu*_​). The values of *K*_*n*_ and *A*_*pu*_ are chosen according to the prevailing system operating conditions to ensure reliable and accurate relay operation.

Figure 2(c) and (d) illustrate the variation of tripping time with respect to the auto-alienation index (*A*_*Auto*_) for the proposed *T*_*op1*_ and *T*_*op2*_ characteristics. From these figures, the following observations can be drawn:

1. Alienation index behavior: The running index (*A*_*Auto*_) increases sharply during fault events, confirming the relay’s sensitivity to abnormal conditions.
2. Inverse time response: The corresponding tripping time (*T*_*op1*_​​ or *T*_*op2*_) decreases as the alienation index (*A*_*Auto*_) rise, demonstrating the expected inverse relationship between the alienation coefficient and operating time.
3. Relay responsiveness: The tripping curve indicates that the proposed algorithm reacts rapidly to severe alienations while maintaining security against spurious trips under minor alienations.
4. Impact of settings: The slope of the tripping curve is governed by the time multiplier (*K*_*m*_ or *K*_*n*_) and pickup threshold (*A*_*pu*_), ensuring adaptability to prevailing system operating conditions.
5. Application of tripping curves: The inverse *A*_*Auto*_–time characteristics provide a practical tool for tuning relay response, enabling coordinated protection settings across different operating scenarios.

##### Comparative analysis of the two tripping-time characteristics

This subsection presents a comparative analysis of the two proposed tripping-time characteristics, namely *T*_*op1*_ and *T*_*op2*_, both formulated based on the auto-alienation index (*A*_*Auto*_) and the pickup value (*A*_*pu*_), while employing different time multiplier constants (*K*_*m*_ and *K*_*n*_). Figure 2(e) shows both tripping-time characteristics (*T*_*op1*_ and *T*_*op2*_) as functions of the auto-alienation index (*A*_*Auto*_). The analysis of the two curves leads to the following key technical observations:

1. The *T*_*op1*_ characteristic exhibits a highly nonlinear inverse-time behavior. As (*A*_*Auto*_) approaches the pickup value (*A*_*pu*_), the denominator (1 - (A_pu_/A_auto_)) tends toward zero, the tripping time increases sharply, resulting in a sharp increase in tripping time. This behavior indicates the presence of a vertical asymptote near the threshold condition and reflects high sensitivity to small deviations around (*A*_*pu*_). Consequently, T_op1_ is particularly effective in distinguishing marginal operating conditions from fault scenarios.
2. For higher values of (*A*_*Auto*_), the T_op1_ curve decreases rapidly and then gradually stabilizes, resulting in faster tripping times under severe conditions (i.e., larger deviation from the pickup value).
3. The *T*_*op2*_ characteristic demonstrates a smooth and monotonically decreasing inverse relationship with (*A*_*Auto*_), without any singularity within the operating range. This ensures stable and predictable performance across all conditions.
4. Compared to *T*_*op1*_, the *T*_*op2*_ curve provides relatively higher tripping times at lower values of (*A*_*Auto*_), but converges toward lower values as (*A*_*Auto*_) increases, indicating improved responsiveness under stronger system disturbances.
5. The difference in curvature between *T*_*op1*_ and *T*_*op2*_ highlights their complementary roles: *T*_*op1*_ offers high sensitivity near the pickup threshold, whereas *T*_*op2*_ ensures stable and gradual operation over a wider range of (*A*_*Auto*_).
6. These characteristics collectively enable flexible coordination of protection settings, allowing the proposed scheme to balance sensitivity and stability under varying operating conditions.
7. In contrast, the *T*_*op2*_ characteristic follows a smooth inverse proportional relationship with respect to (*A*_*Auto*_), without exhibiting any singularity within the defined operating range (*A*_*Auto*_ > *A*_*pu*_). This ensures stable and predictable tripping behavior across all operating conditions. The absence of abrupt changes in tripping time makes Top2 more suitable for applications requiring consistent performance and reduced sensitivity to minor fluctuations.
8. From a comparative perspective, Top1 provides faster tripping for higher values of (*A*_*Auto*_), corresponding to severe system disturbances, while maintaining high sensitivity near the pickup threshold. However, its steep gradient near (*A*_*pu*_) may require careful tuning to avoid excessive delays under near-threshold conditions. On the other hand, *T*_*op2*_ offers a more gradual and uniform response, with relatively higher tripping times at lower values of (*A*_*Auto*_), but improved stability and robustness over a wider operating range.
9. The complementary characteristics of *T*_*op1*_ and *T*_*op2*_ enable flexible coordination in protection system design. Specifically, *T*_*op1*_ can be utilized for sensitive detection and rapid response under critical conditions, whereas *T*_*op2*_ can be employed to ensure stable and reliable operation under normal and moderately disturbed conditions. This coordinated approach enhances the overall performance of the proposed protection scheme by balancing sensitivity, selectivity, and stability.

Overall, the comparative analysis demonstrates that the integration of both tripping-time characteristics provides a robust and adaptable framework for fault detection and system protection in practical applications.

### Protection mechanism

**Fig. 3:**
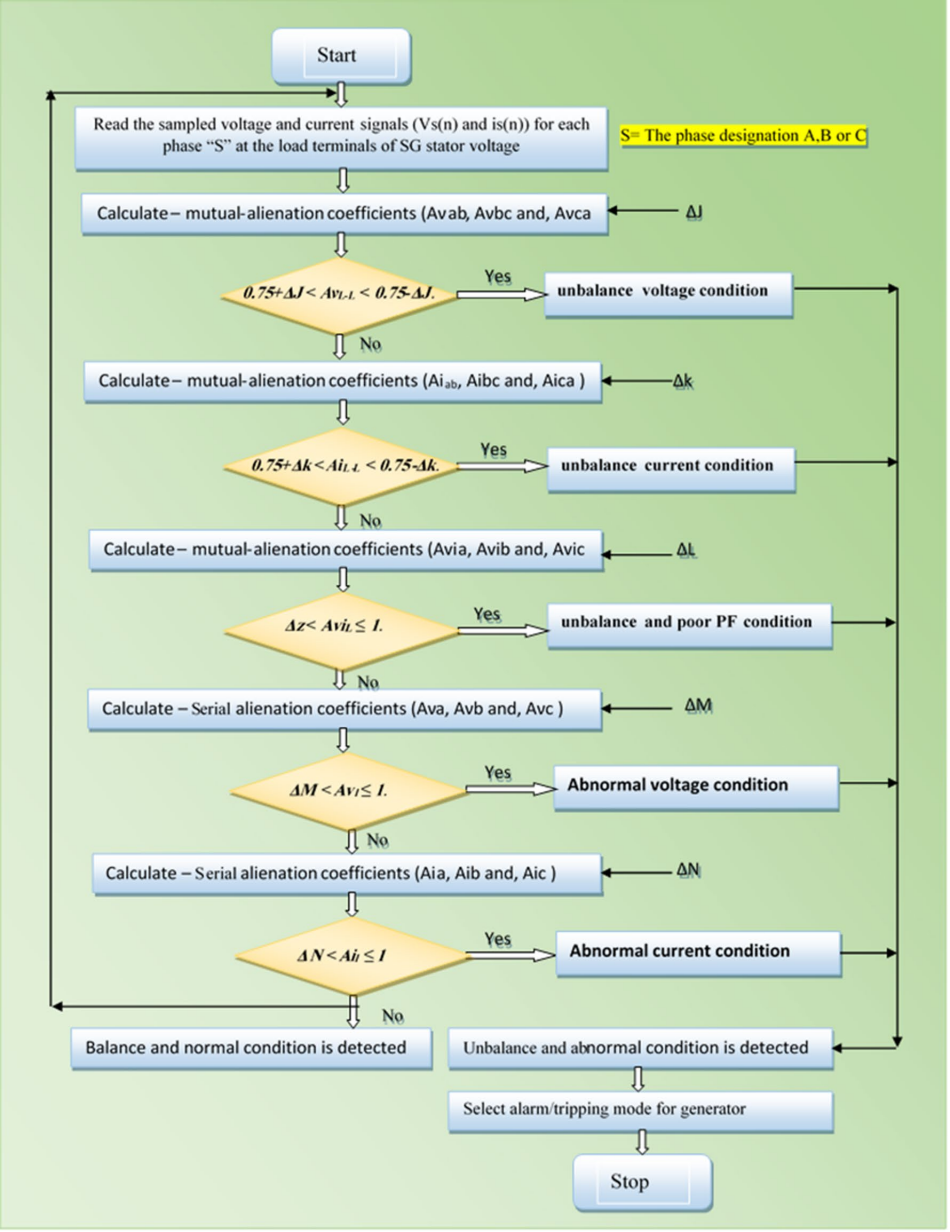
Flowchart of the alienation-based digital protection scheme for synchronous generators.

The proposed protection algorithm, which is based on fifteen alienation factors derived from correlation coefficients, is illustrated in the flowchart shown in Fig. 3. The protection method is implemented as follows:

- 1. Measure the three-phase voltages and currents at the load terminals of the synchronous machine stator windings for phases A, B, and C.
- 2. Determine the total number of samples per cycle and the number of samples per dataset used in the method.
- 3.Select the numerical values of the alienation deviations.
- 4. Run Module 1:
  - Calculate the cross-alienation coefficients between each pair of phase voltages.
  - Detect and assess voltage imbalance conditions using these coefficients and verify the conditions enumerated in Table 1.

- 5. Run Module 2:
  - Compute the cross-alienation coefficients between each pair of phase currents.
  - Detect and evaluate current imbalance conditions using these coefficients and attain the rules given in Table 1.

- 6. Run Module 3:
  - Calculate the cross-alienation coefficients between each phase voltage and its corresponding current.
  - Detect and estimate power factor disturbances using these coefficients and satisfy the conditions presented in Table 1.

- 7. Run Module 4:
  - Compute the auto-alienation coefficients for each phase voltage.
  - Detect and measure voltage disturbances using these coefficients and perform the rules involved in Table 1.

- 8. Run Module 5:
  - Quantify the auto-alienation coefficients for each phase current.
  - Detect and measure current disturbances using these coefficients and achieve the conditions listed in Table 1.

- 9. Execute the corresponding action for the electrical equipment according to the following conditions:
  - Initiate a trip for the generating unit in case of detected fault disturbances or impermissible imbalances in the voltage or current waveforms,
  - Trigger an alarm for permissible voltage or current imbalances, or
  - Maintain a restraining action during normal and balanced operation of the generator voltage and current waveforms.

## Tested generator prototype

**Fig. 4:**
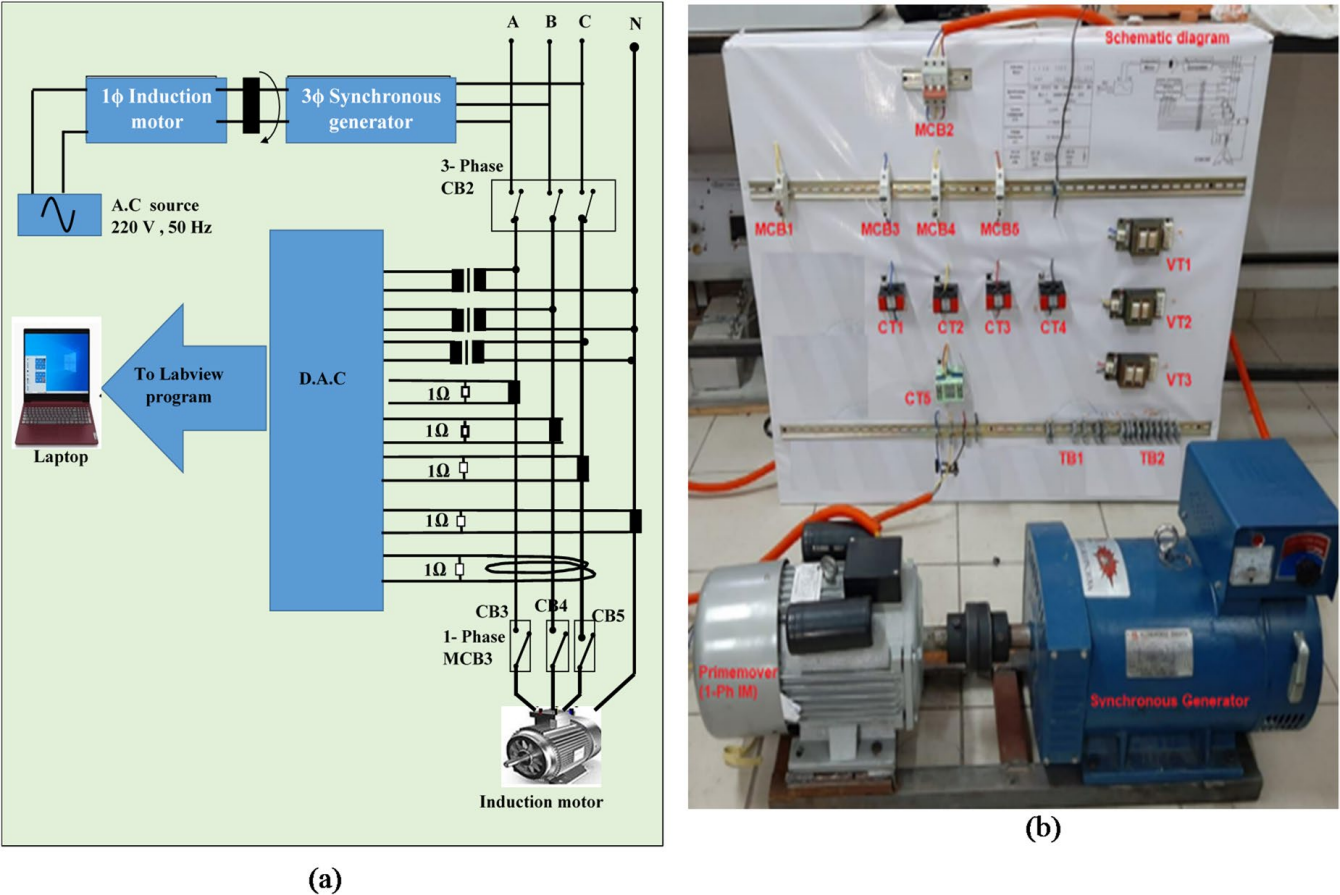
Experimental model of a motor-generator set under investigation: (a) Executive electrical circuit, and (b) Practical electrical circuit.

Practically, a Motor-Generator (M-G) set is a composite system consisting of a motor and a generator mechanically coupled through a common shaft. It is used to convert electrical power from one form to another, primarily converting electrical power into mechanical torque, which is then reconverted to electrical power by the generator. In the configuration shown in Fig. 4(a), electrical power is supplied to the motor, causing its shaft to rotate the generator rotor. Consequently, the generator produces electrical output power while the intermediate power flow between the motor and generator is mechanical. In this study, the motor-generator set is utilized for various power conversion applications:

1. Single-phase AC voltage to three-phase AC voltage.
2. Fixed AC voltage to variable or regulated AC voltage.
3. Alternating power at one frequency to alternating power at another frequency.
4. This is achieved using a single-phase AC induction motor coupled with a three-phase AC synchronous generator with variable excitation voltage.

The practical model validates the performance of the proposed protection system for the three-phase synchronous generator. The experimental setup consists of a three-phase synchronous generator mechanically coupled via a single-phase induction motor, connected to a three-phase load at the SG terminals, as shown in Fig. 4(b). Three-phase voltage and current signals are measured using three voltage transformers (VT1, VT2, and VT3) and three current transformers (CT1, CT2, CT3), respectively, located at the SG load ends. Additionally, CT4 measures neutral current, and CT5 measures residual current of the three phases. The system parameters are summarized in Table 2. The complete power system model and all associated testing procedures have been conducted at the Electrical Power Laboratory, Misr University for Science and Technology (MUST), Egypt.

The three-phase voltage and current measurements are converted into discrete data via a Data Acquisition Card (DAC) for processing in the LABVIEW program. The signals are digitized at a sampling frequency of 2.5 kHz (sampling time of 0.4 milliseconds), with 50 samples per cycle. The intelligent digital relay is emulated using a personal computer alongside the DAC. The DAC device, The National Instruments USB-6009 device is employed to convert analog signals into digital form and operates in differential mode. Extensive experimental tests were performed to simulate various abnormal and unbalanced conditions, including series and shunt faults at the synchronous generator terminals, in order to evaluate the responsiveness’ of the proposed protection method.

**Table 2:** The parameter specifications for the components of the system model under test.

System model parameter	Specification	System model parameter	Specification
*Three phase synchronous generator * *(Power supply)*:		*Voltage Transformer (VT)*	
Type	STC-7.5 (Star connection)	VTR	220 V/ 5 V
Rated power	7.5 kW	Rated burden	5 VA
Rated speed	1500 rpm		
Rated line voltage	380 V		
Rated line current	14.2 A		
Rated frequency	50 Hz		
Number of pair poles	2		
Excitation voltage	100 V		
Excitation current	4 A		
Neutral grounding impedance (*R*_*n*_)	Isolated		
*Single phase induction motor (Prime mover)*:		*Miniature Circuit Breaker (MCB1)*	
Type	YC132SA-4	Phase type	Single phase
Rated power	5 Hp (3.71 kW)	Rated current	63 A
Rated speed (single speed)	1400 rpm	Rated voltage	380 V
Nominal phase voltage	220 V		
Rated current	23 A		
Nominal frequency	50 Hz		
Nominal power factor	*0.80*		
*Three phase induction motor (Load)*:		*Miniature Circuit Breaker (MCB2)*	
Rated power	1.85 kW (Star connection)	Phase type	Three phase
Rated line voltage	400 V	Rated current	20 A
Rated line current	2.7 A	Rated voltage	380 V
Nominal frequency	50 Hz		
Rated speed	1400 rpm		
*Current transformer (CT)*:		*Miniature Circuit Breakers (MCB3 MCB4 * *and MCB5)*	
CTR	200/5	Phase type	Single phase
Rated burden	5 VA	Rated current	20 A
CT burden	1 Ω	Rated voltage	380 V

## Experimental case studies

The experimental prototype is configured as shown in Fig. 4(b), where the generator under test is mechanically driven by a single-phase induction motor supplied from a 220 V source. The generator output is connected to a Data Acquisition Card (DAC), model NI USB-6009, via voltage transformers (200/7) and current transformers (200/5). The LABVIEW program, along with NI-DAQmx driver compatible with Windows Vista/XP/2000/7, is used to interface with the NI USB-6009 for data acquisition and processing. To evaluate the proposed protection technique under abnormal and unbalanced operating conditions of the three-phase synchronous generator, the DAC and LABVIEW software execute the algorithm in real time. The dataset size corresponds to one electrical cycle (N_w_ = N_s_ = 50 samples/cycle), with a full display time of 0.2 milliseconds. This results in a total of 500 samples (N_t_) per display interval.

The following tests present experimental results for various cases, including normal operation, unbalanced conditions, and different fault types. Table 2 lists the pre-fault operating conditions of the practical model, while Appendix 1 provides the input parameters for the alienation-based proposed protection algorithm.

Table 3 involves the wave colors of the measured and computed quantities.

**Table 3:** The wave colors of the measured and computed quantities.

Quantity designation	White color	Red color	Green color
Voltage wave	v_a_	v_b_	v_c_
Current wave	i_a_	i_b_	i_c_
Mutual-alienation coefficient between phase voltage signals	Av_ab_	Av_bc_	Av_ca._
Mutual-alienation coefficient between phase current signals	Ai_ab_	Ai_bc_	Ai_ca._
Mutual-alienation coefficient between phase voltage and current signals	Avi_a_	Avi_b_	Avi_c_
Serial-alienation coefficient for phase voltage signal	Av_a_	Av_b_	Av_c_
Serial-alienation coefficient for phase current signal	Ai_a_	Ai_b_	Ai_c_

Note: Serial- and mutual-alienation coefficients correspond to auto- and cross-alienation coefficients, respectively.

### Discussion of experimental findings

1. **Scenario 1: Currents unbalance of 20%**

Table 4 presents the RMS values of the three-phase voltages and currents, measured using a digital multimeter, while the generator operates under current unbalance condition (fault-free).

**Table 4:** Measured electrical quantities in the case of current unbalance (fault-free).

The 3ϕ RMS primary currents	I_a_	I_b_	I_c_	The 3ϕ primary voltages, V_a_ ≈ V_b_ ≈ V_c_
Reading of primary currents	2.7 A	2.1 A	2.5 A	230 V

The peak values of 3φ secondary current = (I_L_ / CTR) *1.41	*i*_*as*_	*i*_*bs*_	*i*_*cs*_	The peak values of 3ϕ secondary voltages *v*_*as*_ ≈ *v*_*bs*_ ≈ *v*_*cs*_
Reading of secondary currents	0.095 A	0.074 A	0.088 A	7.37 V

**Fig. 5:**
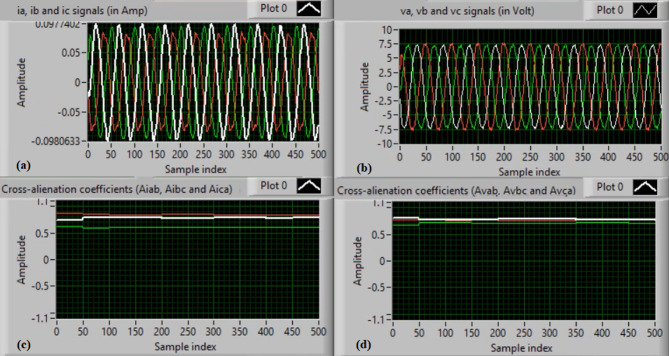
Experimental results for scenario 1. (a) Three phase currents, (b) Three phase voltages, (c) Three phase mutual-alienation coefficients calculated for three phase current signals, and (d) Three phase mutual-alienation coefficients calculated for three phase voltage signals.

Figure 5(a) and (b) show the waveforms of the three-phase current and voltage under normal operation (i.e., without faults). Figure 5(c) and (d) present the computed mutual-alienation coefficients for the three-phase current and voltage signals, respectively. In normal operation, the mutual-alienation coefficients for both current and voltage are approximately + 0.75, indicating an acceptable imbalance in the current/voltage curves caused by slight inaccuracies in the current transformers.

**Fig. 6:**
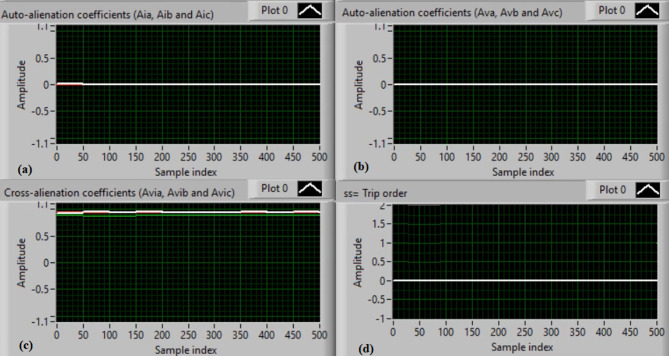
Experimental results for scenario 1 (Continued)(a) Three phase serial-alienation coefficients calculated for three phase current signals, (b) Three phase serial-alienation coefficients calculated for three phase voltage signals, (c) Three phase mutual-alienation coefficients calculated between three phase voltage and current signals, and(d) Tripping signal for scenario 1.

Figure 6(a) and (b) illustrate the serial-alienation coefficients for the three-phase current and voltage signals, respectively, both having a value of zero. It is observed that the sudden variations in the running auto-alienation indices (*Av*_*a*_, *Av*_*b*_, *Av*_*c*_, *Ai*_*a*_, *Ai*_*b*_, and *Ai*_*c*_) for the phase current and voltage signals remain below the specified pickup threshold (0.1), as illustrated in Fig. 6(a) and 6(b). Figure 6(c) illustrates that the mutual-alienation coefficients between the voltage and current signals of the same phase remain nearly constant at + 0.75. Consequently, no disconnection command is issued, as shown in Fig. 6(d). In this case, the fifteen alienation coefficients consistently confirm correct system operation, with the blocking regions of the operating characteristics maintaining stable values.

**(2) Scenario 2: A-phase series fault (MCB**_**3**_ **opening)**

**Fig. 7:**
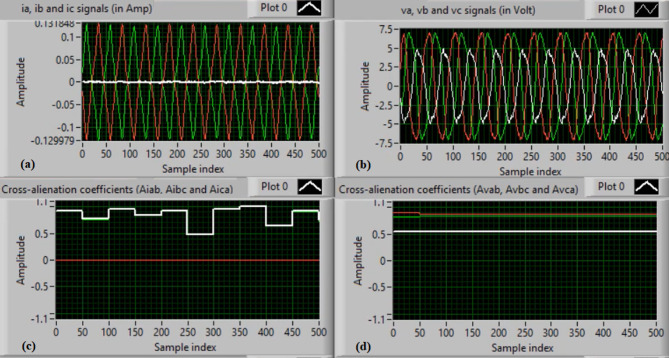
Experimental results for scenario 2. (a) Three phase currents, (b) Three phase voltages, (c) Three phase mutual-alienation coefficients calculated for three phase current signals, and (d) Three phase mutual-alienation coefficients calculated for three phase voltage signals.

**Fig. 8:**
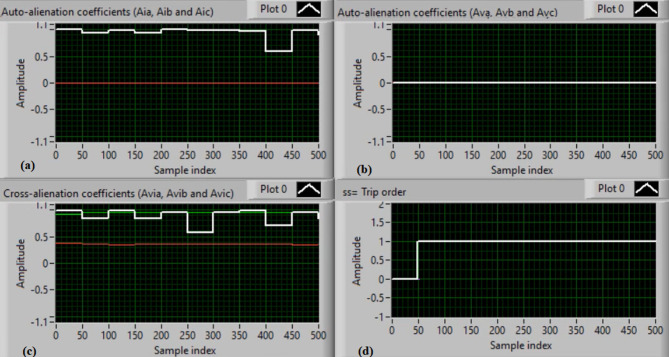
Experimental results for scenario 2 (Continued). (a) Three phase serial-alienation coefficients calculated for three phase current signals, (b) Three phase serial-alienation coefficients calculated for three phase voltage signals, (c) Three phase mutual-alienation coefficients calculated between three phase voltage and current signals, and (d) Tripping signal for scenario 2.

In this case, the phase “A” is manually open-circuited at sample time zero, while all other conditions remain identical to those in Case 1. Figure 7(a) and (b) show the three-phase current and voltage waveforms under this A-phase series fault condition. Figure 7(c) and (d) illustrate the mutual-alienation coefficients between each pair of phase currents and voltages, respectively, where significant deviations are observed as a result of the phase-A open circuit and the pronounced unbalance in the VT and CT signals. Figure 8(a) and (b) depict the serial-alienation coefficients for the three-phase currents and voltages, respectively, while Fig. 8(c) shows the mutual-alienation coefficients between each phase voltage and its corresponding phase current. Finally, Fig. 8(d) displays a high trip-order value, confirming successful detection of the A-phase series fault.

**(3) Scenario 3: C-phase series fault (MCB**_**5**_ **opening)**

**Fig. 9:**
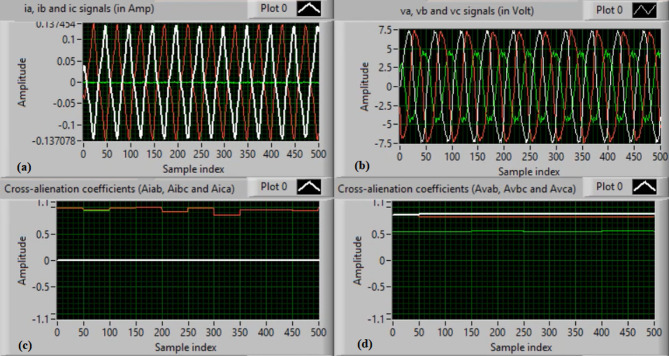
Experimental results for scenario 3. (a) Three phase currents, (b) Three phase voltages, (c) Three phase mutual-alienation coefficients calculated for three phase current signals, and(d) Three phase mutual-alienation coefficients calculated for three phase voltage signals.

**Fig. 10:**
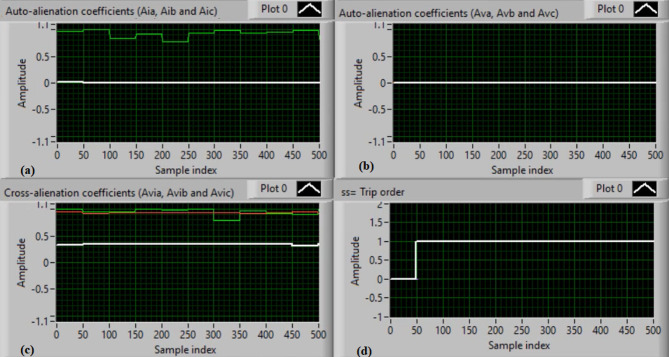
Experimental results for scenario 3 (Continued). (a) Three phase serial-alienation coefficients calculated for three phase current signals, (b) Three phase serial-alienation coefficients calculated for three phase voltage signals, (c) Three phase mutual-alienation coefficients calculated between three phase voltage and current signals, and (d) Tripping signal for scenario 3.

In this case, the phase “C” is manually open-circuited at sample time zero, while all other conditions remain identical to those in Case 1. Figure 9(a) and (b) show the three-phase current and voltage waveforms under the C-phase series fault condition. Figure 9(c) and (d) present the mutual-alienation coefficients between each pair of phase currents and between each pair of phase voltages, reactively, where significant variations are observed due to the open circuit in the phase C and the pronounced unbalance in the current and voltage waveforms. Figure 10(a) and (b) depict the serial-alienation coefficients for the three-phase currents and voltages, respectively. Figure 10(c) shows the mutual-alienation coefficients between each phase voltage and its corresponding phase current. Finally, Fig. 10(d) displays a high trip-order value, confirming detection of the C-phase series fault.

**(4) Scenario 4: A-C-phases series fault (MCB**_**3**_ **and MCB**_**5**_ **opening)**

**Fig. 11:**
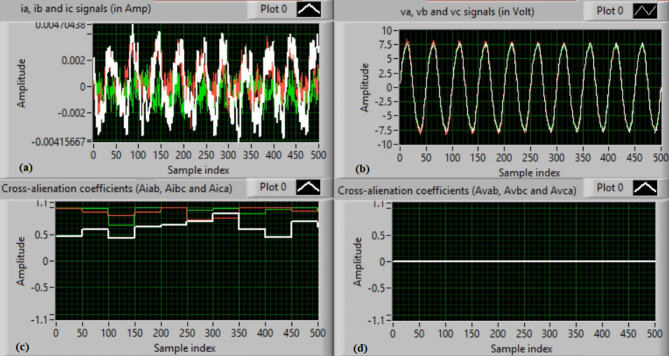
Experimental results for scenario 4. (a) Three phase currents, (b) Three phase voltages, (c) Three phase mutual-alienation coefficients calculated for three phase current signals, and (d) Three phase mutual-alienation coefficients calculated for three phase voltage signals.

**Fig. 12:**
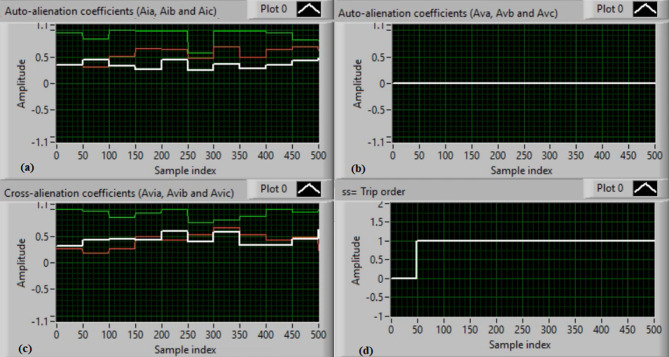
Experimental results for scenario 4 (Continued). (a) Three phase serial-alienation coefficients calculated for three phase current signals, (b) Three phase serial-alienation coefficients calculated for three phase voltage signals, (c) Three phase mutual-alienation coefficients calculated between three phase voltage and current signals, and (d) Tripping signal for scenario 4.

In this case, both phases “A” and “C” are manually open-circuited at sample time zero. Figure 11(a-b) depict the three-phase currents and voltages, respectively, at the instants of fault occurrence and clearing. The manual open-circuit of the two phases introduces a time delay between the current and voltage signals of the faulty phases, which is equivalent to a DL series fault followed by a single line (SL) series fault. Figure 11(c-d) present the mutual-alienation coefficients between each pair of phase currents and between each pair of phase voltages, respectively. Figure 11(c) reveals considerable variations caused by the A–C phase open circuits and the pronounced imbalance in the current waveforms. In contrast, Fig. 11(d) shows no variations in the mutual-alienation coefficients of the voltage waveforms. Figure 12(a) shows the serial-alienation coefficients for the three-phase currents, highlighting significant variations during the fault period. Whereas, the serial-alienation coefficients of the voltage waveforms remain unchanged, as shown in Fig. 12(b). Figure 12(c) illustrates the mutual-alienation coefficients between each phase voltage and its corresponding current, while Fig. 12(d) shows the trip command, indicating that the algorithm successfully detected the series fault condition in both phases “A” and “C”. In this scenario, all alienation coefficients indicate the fault condition, with their values remaining stable within the tripping zones of the developed characteristic curves.

**(5) Scenario 5: SLN (C-N) shunt fault**

**Fig. 13:**
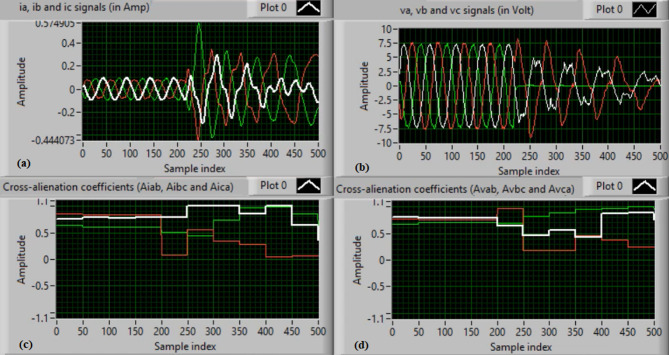
Experimental results for scenario 5. (a) Three phase currents, (b) Three phase voltages, (c) Three phase mutual-alienation coefficients calculated for three phase current signals, and (d) Three phase mutual-alienation coefficients calculated for three phase voltage signals.

In this test, the fault type is an SLN (C-N) shunt fault, located at the load terminals of the synchronous generator under test. Figure 13(a-b) show the three-phase currents and voltages, respectively, at the instant of fault occurrence. As shown in Fig. 13(a), the fault initiates at approximately sample 225. The voltage and current waveforms remain sinusoidal and near nominal values during the first five cycles, but become distorted and change in magnitude during the fault period. Specifically, the instantaneous current values increase, while the three-phase voltage values decrease relative to their nominal levels. During the fault, the peak values of the three-phase secondary voltage signals drop below 7.0 V, whereas the peak values of the three-phase secondary current signals exceed 0.2 A. Figure 13(c) shows the calculated three-phase mutual-alienation coefficients between each pair of current signals, and Fig. 13(d) depicts the mutual-alienation coefficients between each pair of voltage signals. The coefficients vary between 0.0 and 1.0 throughout the fault period.

**Fig. 14:**
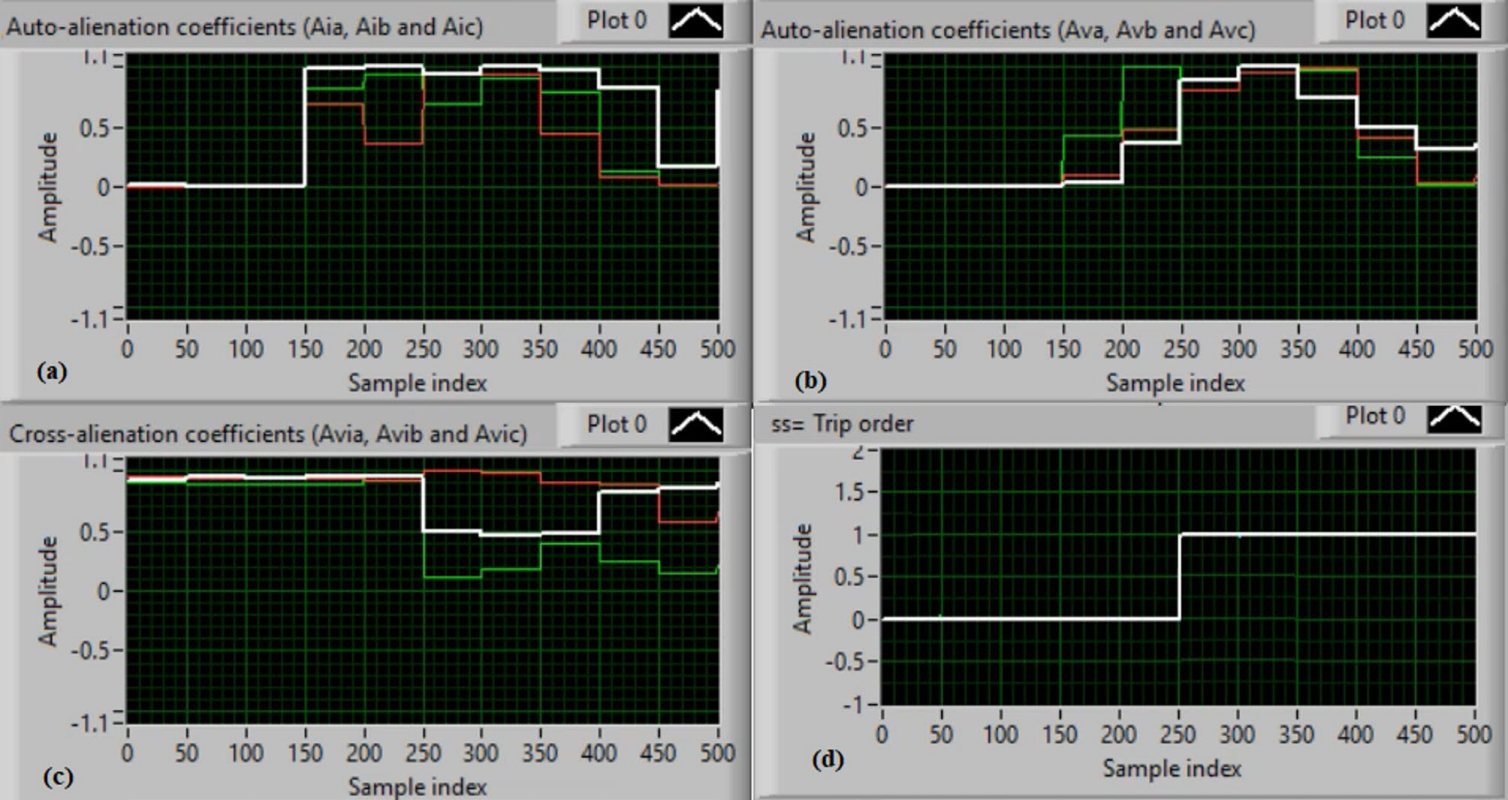
Experimental results for scenario 5 (Continued). (a) Three phase serial-alienation coefficients calculated for three phase current signals, (b) Three phase serial-alienation coefficients calculated for three phase voltage signals, (c) Three phase mutual-alienation coefficients calculated between three phase voltage and current signals, and(d) Tripping signal for scenario 5.

Figure 14(a) shows the three-phase serial-alienation coefficients computed for each phase current signal, while Fig. 14(b) depicts the three-phase serial-alienation coefficients calculated for each phase voltage signal. As illustrated in Fig. 14(a-b), all six serial-alienation coefficients remain steady during normal operation (i.e., the first three cycles) and rise sharply at the onset of the fault. Figure 14(c) presents the three-phase mutual-alienation coefficients calculated between each phase voltage and its corresponding current signal. These coefficients are nearly constant and close to + 0.8 under normal operating conditions, but vary rapidly from + 1.0 to + 0.2 during the fault period. Figure 14(d) displays the tripping signal, which reaches a high value of + 1 in response to the SLN shunt fault. In this case, all alienation coefficients confirm the fault condition, remaining within the tripping zones of the closed-operating characteristics. These experimental results demonstrate that the proposed protection algorithm is accurate, fast, and reliable in detecting abnormal or unbalanced conditions in three-phase voltage and current signals.

**(6) Scenario 6: SLN (B-N) shunt fault**

**Fig. 15:**
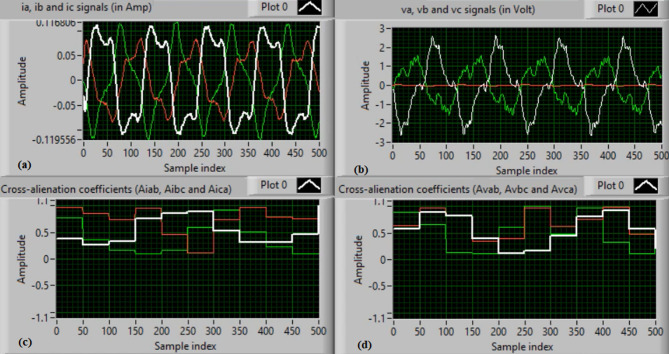
Experimental results for scenario 6. (a) Three phase currents, (b) Three phase voltages, (c) Three phase mutual-alienation coefficients calculated for three phase current signals, and (d) Three phase mutual-alienation coefficients calculated for three phase voltage signals.

**Fig. 16:**
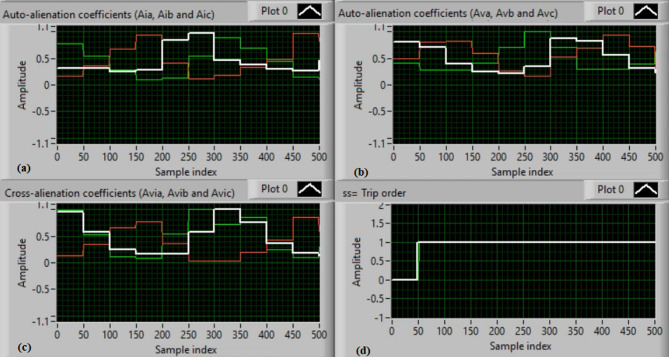
Experimental results for scenario 6 (Continued). (a) Three phase serial-alienation coefficients calculated for three phase current signals, (b) Three phase serial-alienation coefficients calculated for three phase voltage signals, (c) Three phase mutual-alienation coefficients calculated between three phase voltage and current signals, and (d) Tripping signal for scenario 6.

In this test, the fault type is an SLN (B-N) shunt fault, located at the load terminals of the synchronous generator under investigation. Figure 15(a-b) show the three-phase currents and voltages at the moment of fault inception, which occurred at sample zero. During this short circuit, the voltage of the faulted phase drops to nearly zero, while the maximum current reaches approximately 0.8 A. Figure 15(c) presents the three-phase mutual-alienation coefficients computed between each pair of phase currents, which vary significantly during the fault period. Similarly, Fig. 15(d) illustrates the three-phase mutual-alienation coefficients calculated between each pair of phase voltages, also showing non-constant values during the fault. These coefficients fluctuate from + 0.25 to + 1.0 during the fault interval time. Figure 16(a-b) depict the three-phase serial-alienation coefficients for the currents and voltages, respectively, while Fig. 16(c) shows the mutual-alienation coefficients between each phase voltage and its corresponding current. Figure 16(d) displays the tripping signal, which reaches a high value of + 1 for the SLN shunt fault. These results confirm that all alienation coefficients successfully indicate the fault condition, remaining within the tripping zones of the quadratic-operating curves.

**(7) Scenario 7: DL (A-B) shunt fault with CT saturation**

**Fig. 17:**
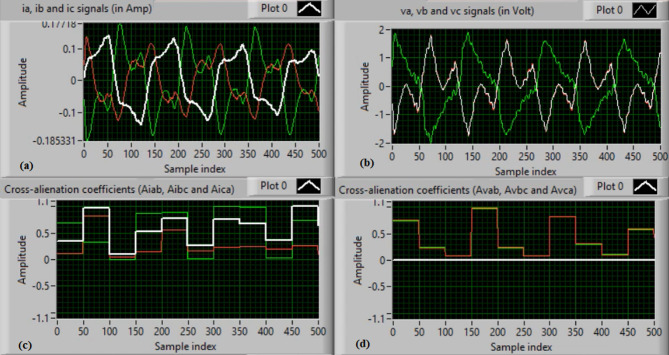
Experimental results for scenario 7. (a) Three phase currents, (b) Three phase voltages, (c) Three phase mutual-alienation coefficients calculated for three phase current signals, and (d) Three phase mutual-alienation coefficients calculated for three phase voltage signals.

**Fig. 18:**
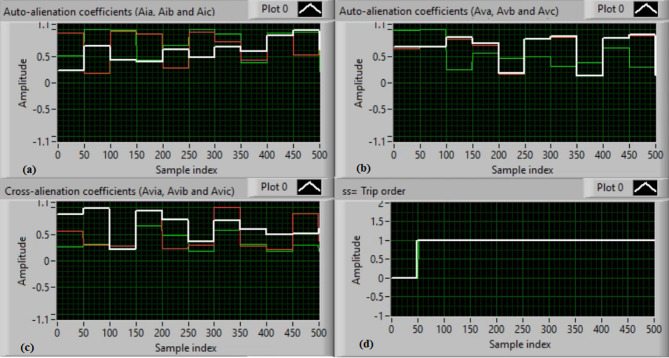
Experimental results for scenario 7 (Continued). (a) Three phase serial-alienation coefficients calculated for three phase current signals, (b) Three phase serial-alienation coefficients calculated for three phase voltage signals, (c) Three phase mutual-alienation coefficients calculated between three phase voltage and current signals, and (d) Tripping signal for scenario 7.

In this case, both phases “A” and “B” are short-circuited. Figure 17(a-b) show the three-phase currents and voltages, respectively. Figure 17(a) indicates that the fault initiates at approximately sample zero. The mutual-alienation coefficients between each pair of phase currents are illustrated in Fig. 17(c), while the mutual-alienation coefficients between each pair of phase voltages are depicted in Fig. 17(d). Figures 18(a-b) present the serial-alienation coefficients for the three-phase currents and voltages, highlighting significant changes in coefficient values at the instant of fault occurrence. Figure 18(c) shows the mutual-alienation coefficients between each phase voltage and its corresponding current, with clearly distinguished values for the three phases. Figure 18(d) displays the tripping signal in response to the DL (A-B) shunt fault. All coefficients range from + 0.0 to + 1.0 during the fault condition accompanied by CT saturation. These results demonstrate that all alienation coefficients accurately indicate the fault condition, with their values concentrated within the tripping zones of the quadratic-characteristic curves.

Case Studies 1, 5, and 7 present illustrative results demonstrating that tripping is governed by the crossing of at least one coefficient over the predefined threshold, rather than by the completion of a full cycle. The nearly constant ~ 50-sample trip time observed across the remaining experiments is a direct consequence of the sliding-window computation adopted in the real-time implementation. Specifically, the alienation coefficient is evaluated at every sampling instant but over a moving window whose length corresponds to one fundamental cycle (50 samples). Consequently, the earliest instance at which a sudden disturbance can be detected occurs upon completion of the first full window containing post-fault samples.

**(8) Scenario 8: DL (A-C) shunt fault with CT saturation**

**Fig. 19:**
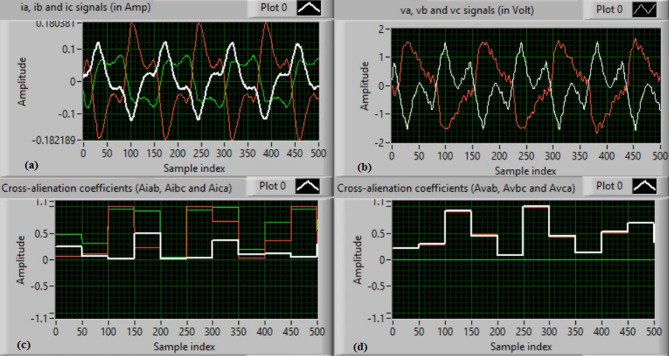
Experimental results for scenario 8. (a) Three phase currents, (b) Three phase voltages, (c) Three phase mutual-alienation coefficients calculated for three phase current signals, and(d) Three phase mutual-alienation coefficients calculated for three phase voltage signals.

**Fig. 20:**
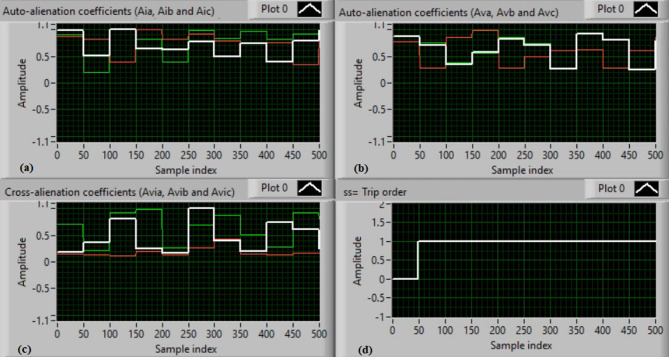
Experimental results for scenario 8 (Continued). (a) Three phase serial-alienation coefficients calculated for three phase current signals, (b) Three phase serial-alienation coefficients calculated for three phase voltage signals, (c) Three phase mutual-alienation coefficients calculated between three phase voltage and current signals, and (d) Tripping signal for scenario 8.

In this case, phases “A” and “C” are short-circuited, with instrumentation CTs and VTs experiencing saturation extent, as shown in Fig. 19(a-b). As shown in Fig. 19(a), the fault occurs at the beginning of the sampling window (approximately sample zero). Despite the CT saturation, the mutual-alienation coefficients successfully detect the abnormal/unbalanced conditions between each pair of phase currents (as seen in Fig. 19(c)) and each pair of phase voltages (as observed in Fig. 19(d)). Figure  20(a-b) display the serial-alienation coefficients for the three-phase currents and voltages, respectively. Figure 20(c) illustrates the mutual-alienation coefficients between each phase voltage and its corresponding current, showing clear deviations from normal operation, indicating the detection of a fault/unbalance condition. During the fault condition with CT saturation, all coefficients exhibit fluctuations ranging from + 0.0 to + 1.0.The trip signal is issued to the generator circuit breaker, as shown in Fig. 20(d). These results confirm that all alienation coefficients consistently identify the fault event, with values lying within the tripping zones of the relay characteristics.

**(9) Scenario 9: DL (B-C) shunt fault with CT saturation**

**Fig. 21:**
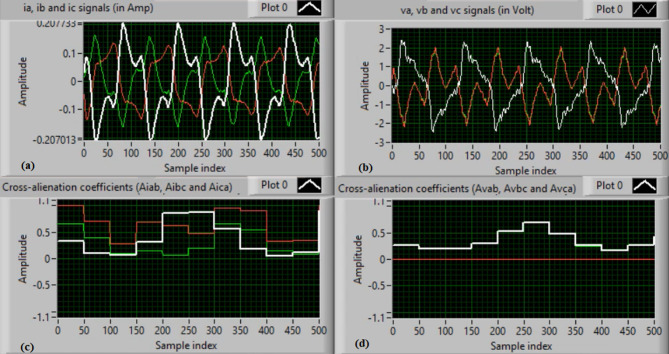
Experimental results for scenario 9. (a) Three phase currents, (b) Three phase voltages, (c) Three phase mutual-alienation coefficients calculated for three phase current signals, and (d) Three phase mutual-alienation coefficients calculated for three phase voltage signals.

**Fig. 22:**
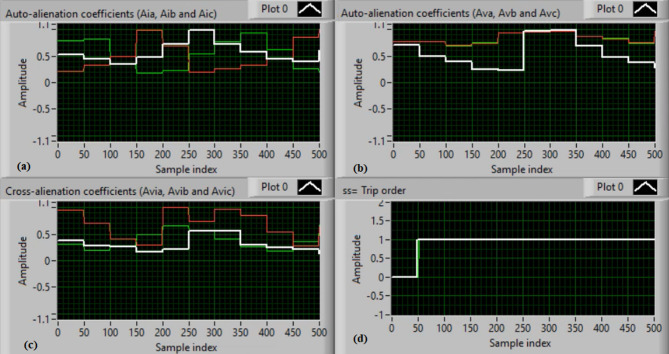
Experimental results for scenario 9 (Continued). (a) Three phase serial-alienation coefficients calculated for three phase current signals, (b) Three phase serial-alienation coefficients calculated for three phase voltage signals, (c) Three phase mutual-alienation coefficients calculated between three phase voltage and current signals, and (d) Tripping signal for scenario 9.

In this case, phases “B” and “C” are also short-circuited, with instrumentation CTs and VTs experiencing saturation, as shown in Fig. 21(a-b). As illustrated in Fig. 21(a), the fault starts near sample zero. Despite the CT saturation, the mutual-alienation coefficients successfully detect the abnormal/unbalanced conditions between each pair of phase currents (as illustrated in Fig. 21(c)) and each pair of phase voltages (as depicted in Fig. 21(d)). Figure 22(a-b) display the serial-alienation coefficients for the three-phase currents and voltages, respectively. Figure 22(c) shows the mutual-alienation coefficients between each phase voltage and its corresponding current, indicating clear deviations from normal operation. All coefficients oscillate between + 0.0 and + 1.0 under the fault condition with CT saturation. These coefficients confirm that the algorithm detected the fault/unbalance condition, and a trip signal has been issued to the machine circuit breaker, as shown in Fig. 22(d).

In this examination, it is evident that all alienation coefficients reliably indicate the presence of a fault, with their values consistently falling within the tripping zones of the closed-characteristics. Comprehensive experimental tests demonstrate that the alienation coefficient values remain stable under normal operation and acceptable unbalance conditions, remaining within the restraining regions of all four tripping characteristics. Conversely, during abnormal conditions—such as series and shunt faults, CT saturation, loss of synchronism, or desynchronization events—most coefficients shift into the tripping regions. These results confirm the effectiveness of the proposed protection system as a backup relay for synchronous generators, using the alienation coefficients derived from three-phase voltage and current measurements to accurately detect and respond to various fault and unbalance scenarios.

### Analysis of experimental findings

Tables 5 and 6 summarize the cross- and auto-alienation analysis for various fault types located at the synchronous generator load terminals. Table 5 presents the quantitative values of the mutual-alienation coefficients, while Table 6 provides the corresponding quantitative values of the serial-alienation coefficients.

Table 5 shows that the mutual-alienation values remain constant during normal operation. When a fault occurs, these values drop to zero and exhibit instability depending on the type of fault. Table 6 shows that the serial-alienation values are zero under normal operation, but become unstable during faults, with variations dependent on the fault type and the affected phase, occurring after 50 samples.

The use of the alienation factor provides several key features:

- High accuracy, speed, and reliability for detecting faults and protecting various electrical components, including generators, transformers, and bus bars, as well as identifying imbalances in electrical networks.
- Adjustable security and sensitivity by predefining the number of datasets and selecting appropriate alienation settings.
- Implementation of five innovative types of alienation coefficients (ΔJ, ΔK, ΔZ, ΔM, and ΔN) with values ranging from 0.0 to 1.0.
- Compatibility with digital protection systems and relays, such as overcurrent relays, differential overcurrent relays, and voltage balance relays, allowing calibration for optimal performance.

**Table 5:** Cross-alienation coefficient values for various fault types located at the synchronous generator load terminals.

Case No.	Case description	Fault-inception sample	Fault-clearance sample	Mutual-alienation for voltage signals			Mutual-alienation for current signals			Mutual-alienation for for voltage and current signals		
				Av_ab_	Av_bc_	Av_ca._	Ai_ab_	Ai_bc_	Ai_ca._	Avi_a_	Avi_b_	Avi_c_
Case 1	No fault with current unbalance of 20%	NA	NA	*0.7–0.75*	*0.7–0.75*	*0.55–0.6*	*0.7–0.75*	*0.75–0.85*	*0.55–0.6*	*0.9-1*	*0.9-1*	*0.9-1*
Case 2	A-phase series fault (MCB3 opening)	0.0	500	**0.85**	**0.55**	**0.85–0.9**	**0.5-1**	**0.0**	**0.5-1**	**0.4**	**1-0.6**	**0.9**
Case 3	C-phase series fault (MCB5 opening)	0.0	500	**0.6**	**0.85**	**0.85**	**0.0**	**0.85-1**	**0.85-1**	**0.8-1**	**0.8-1**	**0.35–0.4**
Case 4	A-C phases series fault	0.0	500	**0.0**	**0.0**	**0.0**	**0.5-1**	**0.5-1**	**0.75-1**	**0.4–0.7**	**0.4–0.5**	**0.7-1**
Case 5	SLN (C-N) shunt fault	250	500	**Not constant 0.5–0.7**	**Not constant 0.1-1**	**Not constant 0.6-1**	**Not constant 0.7-1**	**Not constant 0-0.7**	**Not constant 0.5-1**	**Not constant 0.5-1**	**Not constant 0.5-1**	**Not constant 0.1-1**
Case 6	SLN (B-N) shunt fault	0.0	500	**Not constant** **0.1-1**	**Not constant** **0.4-1**	**Not constant** **0.1-1**	**Not constant** **0.3–0.9**	**Not constant** **0.1–0.9**	**Not constant** **0.1–0.9**	**Not constant** **0.3-1**	**Not constant** **0.1-1**	**Not constant** **0.1–0.9**
Case 7	DL (A-B) shunt fault with CT saturation	0.0	500	**0.0**	**Not constant** **0.1-1**	**Not constant** **0.1-1**	**Not constant** **0.1-1**	**Not constant** **0.1–0.8**	**Not constant** **0-0.8**	**Not constant** **0.2-1**	**Not constant** **0.2-1**	**Not constant** **0.2-1**
Case 8	DL (A-C) shunt fault with CT saturation	0.0	500	**Not constant** **0.2-1**	**Not constant** **0.2-1**	**0.0**	**Not constant** **0-0.5**	**Not constant** **0–1**	**Not constant** **0–1**	**Not constant** **0.2-1**	**Not constant** **0.2-1**	**Not constant** **0.2-1**
Case 9	DL (B-C) shunt fault with CT saturation	0.0	500	**Not constant** **0.2–0.7**	**0.0**	**Not constant** **0.2–0.7**	**Not constant** **0.1–0.8**	**Not constant** **0.1–0.8**	**Not constant** **0.1–0.7**	**Not constant** **0.2–0.6**	**Not constant** **0.2-1**	**Not constant** **0.2–0.6**

Significant values are in [bold and italics].

**Table 6:** Auto-alienation coefficient values for various fault types located at the synchronous generator load terminals.

Case No.	Case description	Serial-alienation for voltage signal			Serial-alienation for current signal			Trip order
		AV_a_	AV_b_	AV_c_	AI_a_	AI_b_	AI_c_	
Case 1	No fault with current unbalance of 20%	*0.0*	*0.0*	*0.0*	*0.0*	*0.0*	*0.0*	*No-trip*
Case 2	A-phase series fault (MCB3 opening)	0.0	0.0	0.0	**0.55-1**	0.0	0.0	High
Case 3	C-phase series fault (MCB5 opening)	0.0	0.0	0.0	0	0.0	**0.8-1**	High
Case 4	A-C phases series fault	0.0	0.0	0.0	**0.4–0.7**	**0.4–0.5**	**0.7-1**	High
Case 5	SLN (C-N) shunt fault	**Not constant ** **0–1**	**Not** **constant** **0–1**	**Not constant** ** 0–1**	**Not** **constant** **0-1**	**Not constant ** **0–1**	**Not constant** ** 0–1**	High
Case 6	SLN (B-N) shunt fault	**Not constant** **0.2–0.8**	**Not** **constant** **0.2–0.9**	**Not constant** **0.3-1**	**Not** **constant** **0.2-1**	**Not constant** **0.2-1**	**Not constant** **0.1–0.8**	High
Case 7	DL (A-B) shunt fault with CT saturation	**Not constant** **0.2-1**	**Not** **constant** **0.2-1**	**Not constant** **0.2-1**	**Not** **constant** **0.2-1**	**Not constant** **0.2-1**	**Not constant** **0.2-1**	High
Case 8	DL (A-C) shunt fault with CT saturation	**Not constant** **0.3-1**	**Not** **constant** **0.3-1**	**Not constant** **0.3-1**	**Not** **constant** **0.3-1**	**Not constant** **0.3-1**	**Not constant** **0.3-1**	High
Case 9	DL (B-C) shunt fault with CT saturation	**Not constant** **0.4-1**	**Not** **constant** **0.6-1**	**Not constant** **0.6-1**	**Not** **constant** **0.4-1**	**Not constant** **0.2-1**	**Not constant** **0.2-1**	High

Significant values are in [bold and italics].

Table 7 presents the estimated operating times calculated using the six serial-alienation coefficients (*Av*_*a*_, *Av*_*b*_, *Av*_*c*_, *Ai*_*a*_, *Ai*_*b*_, and *Ai*_*c*_) for the phase voltage and current signals. The actual relay tripping time (in seconds) is defined as the minimum operating time (*T*_*op1*_) estimated from the six serial-alienation indices.

**Table 7:** Tripping times (*T*_*op1*_) based on auto-alienation coefficients.

Case No.	Case description	T_op_ using serial-alienation for voltage signal (seconds)			T_op_ using serial-alienation for current signal (seconds)			Actual tripping time, T_op1_ (seconds)
		The setting parameters K_m_ and A_pu_ are set to 3.33 and 0.1, respectively.						
		AV_a_	AV_b_	AV_c_	AI_a_	AI_b_	AI_c_	
Case 1	No fault with current unbalance of 20%	*∞*	*∞*	*∞*	*∞*	*∞*	*∞* ***∞***	*∞* ***∞***
Case 2	A-phase series fault (MCB3 opening)	∞	∞	∞	**0.333**	∞	∞	**0.333**
Case 3	C-phase series fault (MCB5 opening)	∞	∞	∞	∞	∞	**0.333**	**0.333**
Case 4	A-C phases series fault	∞	∞	∞	**0.350**	**0.375**	**0.333**	**0.333**
Case 5	SLN (C-N) shunt fault	**0.333**	**0.333**	**0.333**	**0.333**	**0.333**	**0.333**	**0.333**
Case 6	SLN (B-N) shunt fault	**0.343**	**0.338**	**0.333**	**0.333**	**0.333**	**0.343**	**0.333**
Case 7	DL (A-B) shunt fault with CT saturation	**0.333**	**0.333**	**0.333**	**0.333**	**0.333**	**0.333**	**0.333**
Case 8	DL (A-C) shunt fault with CT saturation	**0.333**	**0.333**	**0.333**	**0.333**	**0.333**	**0.333**	**0.333**
Case 9	DL (B-C) shunt fault with CT saturation	**0.333**	**0.333**	**0.333**	**0.333**	**0.333**	**0.333**	**0.333**

Significant values are in [bold and italics].

**Fig. 23:**
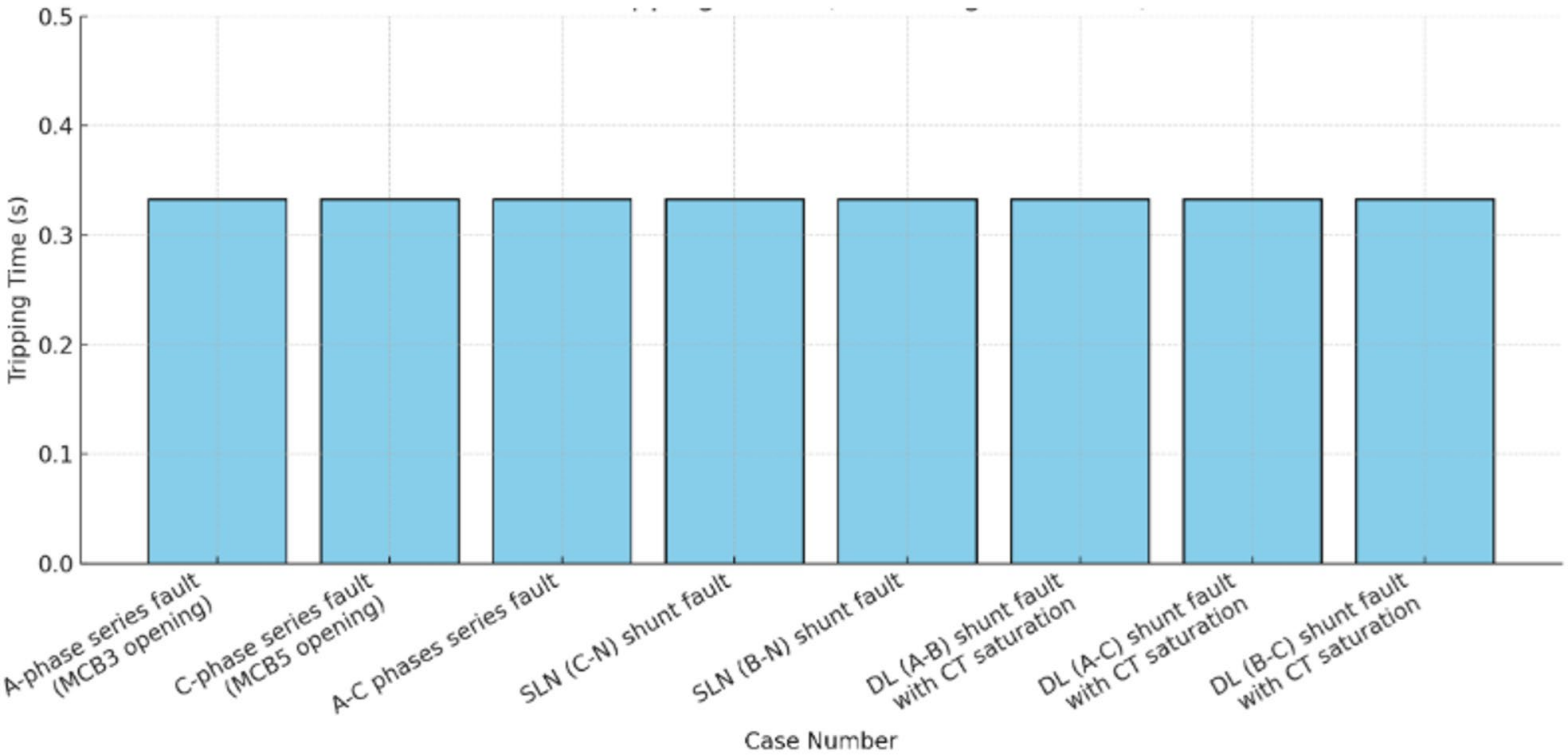
Tripping time (*T*_*op1*_) versus case number.

Figure 23 presents the bar chart of actual tripping times (in seconds) versus case number, with Case 1 (no fault) excluded since its tripping time is ∞ (infinite). For all remaining fault cases, the relay operated at a uniform tripping time of 0.333 s, resulting in equal bar heights across the chart. This demonstrates that, under the given test conditions, the relay provided a consistent and deterministic response to all fault scenarios. Nevertheless, it should be noted that the relay’s operating time is inherently influenced by the type and location of the fault, which may lead to variations in other test settings or practical applications.

## Alienation-based protection scheme features

### Protection characteristics assessment

In this project, the protection system issued a total of 2,410 trip signals, of which 4 events were false trips. The algorithm failed to trip in 5 instances across all tests. Additionally, the protection scheme remained inactive 55 times without any malfunction when operating under normal conditions. Table 8 presents the mathematical models used to evaluate the protection characteristics, including the estimated percentages of dependability (DP), security (SP), reliability (RP), and accuracy (AP) of the algorithm under various fault disturbances, as well as the influence of voltage and current transformer errors^39–42^. The results indicate that the estimated values of DP, SP, RP, and AP all exceed 99.6%.

**Table 8:** Evaluation of the protection characteristics.

Power system state	Number of scenarios	Malfunction times
The total number of trips	2410	4.0 (The protection algorithm issued an incorrect trip in 4.0 scenarios of no-faults due to residual flux and harmonics, resulting from the largest CT saturation degree, which originates from the high- fault current in different cases)
The total number of normal operations	55	0.0
Protection characteristics evaluation	Qt_1_ = The total number of scenarios = 2465	Malfunction times = 4.0
	Qt_2_ = The total number of trips	= 2410
	Qt_3_ = The number of correct trips	= 2406
	Qt_4_ = The number of tripping failures	= 5.0 (The protection algorithm failed to trip in 5.0 scenarios of the faults as a result of the arc fault resistances)
	Qt_5_ = The number of desirable trips = Qt_3_ + Qt_4_	= 2406 + 5 = 2411
	Qt_6_ = The number of incorrect trips = Qt_2_ – Qt_3_	= 2410–2406 = 4
	Qt_7_ = Qt_5_ + Qt_6_ = Qt_2_ + Qt_4_	= 2411 + 4 = 2410 + 5 = 2415
	$\:\%\:DP=\:\frac{\mathrm{Q}\mathrm{t}3\:}{\mathrm{Q}\mathrm{t}5}\times\:100$	= $\:\frac{2406}{2411}$ $\:\times\:\:$100 = $\:99.80\:\%$
	$\:\%\:SP=\:\frac{\mathrm{Q}\mathrm{t}3}{\mathrm{Q}\mathrm{t}2}\times\:100$	= $\:\frac{2406}{2410}\times\:\:$100 = $\:99.83\:\%$
	$\:\%\:RP=\:\frac{\mathrm{Q}\mathrm{t}3}{\:\mathrm{Q}\mathrm{t}7}\times\:100$	= $\:\frac{2406}{2415}\times\:\:$100 = $\:99.63\:\%$
	$\:\%\:AP=\:\frac{\mathrm{Q}\mathrm{t}1-\mathrm{Q}\mathrm{t}4-\mathrm{Q}\mathrm{t}6}{\mathrm{Q}\mathrm{t}1}\times\:100$	= $\:\frac{2465-5-4\:}{2465}\:\times\:\:$100 = 99.63%
	$\:\%\:MR=\:\frac{\mathrm{Q}\mathrm{t}4\:+\:\mathrm{Q}\mathrm{t}6}{\mathrm{Q}\mathrm{t}1}\times\:100$ = = 100% - *AP* %	= $\:\frac{5+4\:}{2465}\:\times\:\:$100 = 0.37%

**Notes**: DP = Dependability Percentage, SP = Security Percentage, RP = Reliability Percentage, AP = Accuracy Percentage, and MR = Malfunction Rate.

Table 9 presents a comparative evaluation of the protection characteristics between the currently submitted work (correlation-based alienation method) and the previously published study (coherence-based alienation method)^31^. The analysis in Table 9 confirms that the correlation-based alienation method achieves improved performance metrics, higher accuracy, and a lower malfunction rate compared to the coherence-based approach^31^.

Furthermore, correlation quantifies the linear relationship between the reference and measured signals in the time domain, making it inherently more sensitive to rapid variations caused by machine faults. In contrast, coherence is a frequency-domain metric that averages signal behavior over a spectral window, which can smooth or mask short-duration disturbances and reduce detection sensitivity under transient conditions. Additionally, the correlation-based formulation exhibits faster computational convergence and does not require windowing or spectral estimation, thereby enhancing responsiveness and reducing numerical uncertainty.

**Table 9:** Comparison of protection characteristics between the correlation and coherence-based alienation methods.

Designation	Correlation-based alienation method (Current study)	Coherence-based alienation method (Previously published article^31^)
The total number of scenarios	**2465**	*270*
The total number of trips	**2410**	*230*
The number of malfunction trip times	**4.0**	*2.0*
The total number of normal operations	**55**	*40*
Malfunction times in the case of normal operations	**0.0**	*0.0*
The total number of the algorithm failed to trip	**5.0**	*2.0*
Protection characteristics evaluation		
Dependability	**99.80%**	*99.13%*
Security	**99.83%**	*99.13%*
Reliability	**99.63%**	*98.28%*
Accuracy	**99.63%**	*98.52%*
Malfunction rate	**0.37%**	*1.48%*

Significant values are in [bold and italics].

#### Sensitivity analysis

A sensitivity analysis was conducted to evaluate the impact of the threshold setting on the performance of the proposed protection scheme. The analysis examined how variations in the threshold influence key performance metrics, including detection accuracy and malfunction rate. Results indicate that lower threshold values increase sensitivity to incipient faults but may lead to a higher malfunction rate due to occasional false tripping under no-fault conditions. Conversely, higher threshold values improve security by reducing false operations, at the expense of slightly delayed or missed fault detection. An optimal threshold region was identified that achieves a balanced trade-off between dependability and security, minimizing the malfunction rate while maintaining high detection accuracy. This analysis confirms that the reported malfunction rate can be further reduced through appropriate threshold tuning without compromising reliable fault detection.

The sensitivity analysis is an important concern in the coordination study.

For coordination pairs in which the backup relays do not meet the required sensitivity, the operation time may be prolonged. Relay sensitivity is directly related to the selected numerical values of the setting deviations: lower setting values result in higher sensitivity. Similarly, shorter data window lengths also increase relay sensitivity.

$${\mathrm{Sensitivity~metric~\boldsymbol{\upalpha}~}}\left| {\frac{1}{{{\mathrm{Setting~deviation}}}}} \right|$$

$${\mathrm{Sensitivity~metric~\boldsymbol{\upalpha}~}}\left| {\frac{1}{{{\mathrm{Data~set~lenghth}}}}} \right|$$

#### Speed analysis

The detection speed can be readily controlled through the predetermined size of the data window used to calculate the alienation coefficients, which is typically less than or equal to one periodic cycle. The window length can be set to one cycle or a fraction thereof, thereby allowing direct control over the fault detection time.

The proposed approach performs detection and assessment functions simultaneously for fault and unbalance events. Specifically, the alienation coefficient quantifies the degree of association between any two signals or data sets, thereby revealing the level of synchrony between them. Consequently, it serves as a suitable estimator for evaluating the association degree and for identifying sudden changes between the two signals or data sets in real time.

#### Fault identification accuracy

A set of novel tripping curves can be developed based on the alienation coefficients computed from voltage and current data for each data window. These curves enable the protection algorithm to operate effectively during fault and unbalance conditions, thereby preventing system damage, while restraining operation under balanced and normal operating states of the power network. The proposed algorithm can readily select appropriate threshold settings for the alienation estimators to distinguish between acceptable and unacceptable imbalance levels, as well as between faulted and normal operating conditions.

A trade-off between speed and accuracy must be considered, as these requirements are inherently conflicting. Faster protection schemes generally exhibit reduced accuracy because shorter decision windows provide less information for reliable decision-making.

#### Malfunction rate (MR)

The Malfunction Rate (MR) of the protection algorithm refers specifically to the percentage of test cases—within a controlled set of laboratory experiments—in which the protection scheme either failed to correctly identify an actual fault condition or issued an incorrect trip under no-fault conditions. Accordingly, this metric represents the fraction of evaluated instances (rather than operating time) that were incorrectly classified during testing, for which the threshold setting failed to produce the expected response—namely, a tripping action for actual fault conditions and a blocking action under no-fault conditions.

In short, the malfunction rate is define as follows: “The ratio of incorrectly classified cases to the total number of evaluated cases across all experimental fault scenarios.”

In this study, the malfunction rate (MR) is mathematically estimated as follows:

*Qt*_*1*_ = The total number of scenarios = 2465,

*Qt*_*4*_ = The protection algorithm failed to trip in 5.0 scenarios of the faults,

*Qt*_*6*_ = The protection algorithm issued an incorrect trip in 4.0 scenarios of no-faults,

*AP* = Accuracy percentage$~=~\frac{{Qt1 - Qt4 - Qt6}}{{Qt1}} \times 100$ = $\frac{{2465 - 5 - 4~}}{{2465}}~ \times ~$100 = 99.63%,

$${\mathrm{Malfunction~Rate}}=~~MR=~\frac{{Qt4~+~Qt6}}{{Qt1}} \times 100~{{\% }}$$ = $$\frac{{5+4~}}{{2465}}~ \times ~$$100% = 0.37%, or

$${\mathrm{Malfunction~Rate}}=~~MR=~100$$ % - $$~AP$$ $$\% ~$$= $$100$$ % - $$~99.63{\mathrm{~}}$$ $$\%$$= 0.37%.

#### Threshold value identification

The threshold values of the alienation coefficients and their allowable deviations were determined experimentally by monitoring the alarm flags and relay response signals under normal operating conditions. The settings in the LabVIEW environment were adjusted to tolerate acceptable levels of voltage and current unbalance, as well as normal power factor conditions at full load. These settings can be readily reconfigured for different experimental setups, depending on the permissible limits of voltage unbalance, current unbalance, and power factor, which represents a key advantage of the proposed algorithm.

During operation, the protection relay compares the calculated alienation coefficients with their corresponding threshold values. In practice, the effects of measurement errors, DC offsets, moderate harmonic distortion, and transient disturbances can be mitigated through appropriate selection of the data window size and alienation setting deviations. Adjusting these parameters allows effective tuning of the protection scheme to meet desired performance requirements, including accuracy, security, sensitivity, stability, and speed. Consequently, the relay decision logic is governed by the calculated alienation coefficients in relation to their predefined thresholds.

#### Scalability analysis

Regarding scalability and the applicability of the proposed protection scheme to larger-capacity generators and different machine types, the alienation-coefficient formulation is fundamentally signal-based and does not depend on parameters that vary with generator size. Consequently, its decision criterion remains valid for medium- and large-scale synchronous generators. In practical implementation, the method can be applied without modifications to measurement hardware (except for standard voltage and current transformers), as it relies on voltage and current signals available at the generator terminals. Sampling rates and window lengths may be adjusted proportionally to the generator’s fundamental frequency, ensuring accurate detection without affecting the core algorithm. This makes the approach readily deployable across a wide range of generator ratings and configurations.

### Alienation-based protection benefits

The developed protection technique, based on alienation derived from the correlation concept, offers the following advantages:

1. Abnormal and unbalanced conditions in the three-phase voltage and current signals can be continuously monitored and identified quickly and accurately using alienation coefficients estimated from the electrical signals.
2. The proposed approach enables the development of new quadratic tripping characteristics based on mutual- and serial-alienation indices, allowing the discrimination between fault and no-fault conditions, the differentiation of unbalanced and balanced states, and the quantification of voltage and current asymmetry severity.
3. An alienation-based tripping-time curve is proposed for the backup protection scheme, ensuring a controlled time-delayed tripping response under fault conditions.
4. The size of the dataset can be tuned to achieve a fast response, typically one cycle or sub-cycle; the window size directly affects the detection time of abnormal and unbalanced conditions.
5. Alienation settings can be adapted based on prevailing power system conditions and the acceptable voltage and current unbalance.
6. The severity of voltage and current deviations can be assessed for all possible contingencies and unbalances using the alienation coefficients, making the method a useful tool for mitigating unbalance effects in power systems.
7. The technique is accurate, reliable, and robust, and it can be applied across generation, transmission, distribution, and utilization systems at various voltage levels. It also provides an effective solution for unbalance detection and assessment in traditional grids, smart grids, and digital substations.
8. Sensitivity and security are adjustable through the proper selection of alienation/correlation deviation thresholds and the length of the correlation dataset.
9. It is independent of the parameter data of power system elements and instrument transformers, such as voltage and current transformers.
10. The proposed algorithm can serve as a foundation for digital fault recorders, protective relays, and voltage/current disturbance detectors.
11. It can be implemented in adaptive protection relays and systems, enabling the adjustment of tripping characteristics for devices such as differential overcurrent relays and voltage-controlled time overcurrent relays.

### Critical comparison

The comparison in Table 10 clearly demonstrates that the proposed alienation/correlation-based protection scheme offers notable advantages over conventional backup relays. By relying on a limited number of bounded alienation/correlation setting values, the proposed method significantly simplifies relay configuration while enabling adaptive tuning under varying operating conditions. The bounded tripping characteristics enhance security and selectivity, whereas the alienation-based time-delay formulation allows fast, including sub-cycle, fault detection compared with RMS-based conventional relays. Furthermore, the proposed scheme integrates multiple protection and assessment functions within a unified algorithmic framework, eliminating the need for data synchronization and additional filtering stages. Its inherent capability to detect CT saturation further improves relay stability during external faults. Overall, the proposed approach achieves higher sensitivity, faster response, and reduced computational complexity, establishing it as a robust and innovative alternative to conventional backup protection methods.

**Table 10:** Comparison between the alienation/correlation-based protection scheme and conventional backup relays.

Item of comparison	Alienation/correlation-based protection scheme	Conventional backup relays
1. Selected setting values	Requires only two setting values of alienation/correlation coefficients for all variables, simplifying relay configuration.	Require various settings for voltage/ current magnitude, phase shift, and time delay
	Allows adaptive tuning of alienation/ correlation settings in response to prevailing power system operating conditions	
2. Tripping characteristics type	Proposes novel bounded tripping curves formulated from alienation/correlation coefficients, constrained within the range [0.0, + 1.0], analogous to per-unit magnitudes	Mostly have open tripping curves without restricted values for voltage and/or current. Only impedance relays as backup for SG have restricted characteristics
3. Tripping time delay (Relay speed)	An alienation-based tripping-time curve is established within the backup protection scheme, providing a systematic time-delayed tripping action during fault conditions	Typically greater than one cycle because conventional methods rely on RMS calculations requiring at least one cycle
	Controllable using the dataset size; can be less than one cycle (sub-cycle), enabling faster relay operation	
4. Multi-algorithms and multi-functions	Can perform multiple protection functions:	Some relays perform only a single protection function.
	- Voltage and current disturbance detection	Others execute multiple algorithms for multi-function protection, requiring different mathematical formulas
	- Voltage and current unbalance detection	
	- Assessment of voltage/current unbalance	
	- Assessment of voltage/current fault disturbances	
	Can also serve as a base for digital synchro-check relays, power factor correctors, and adaptive differential current relays	
5. Data synchronization system	No data synchronization system required.	Some conventional relays require a data synchronization system to ensure accurate and reliable data transmission
6. Relay properties	Simple, accurate, dependable, secure, reliable, sensitive, and stable	Some relays are complex but maintain accuracy, dependability, security, reliability, sensitivity, and stability
7. Criteria simplicity	Simple in maintenance, installation, and operation; simpler systems are generally more reliable	Some conventional systems are complex, making maintenance and installation more difficult
8. Cost of implementation	Requires a Data Acquisition Card (DAC) to convert analog signals into digital form, along with its associated driver software	Requires DAC and its associated driver software
	Implements a single algorithm dedicated to calculating alienation/correlation factors	Some conventional relays process multiple algorithms (RMS calculation, phase angle, relay operating time, and other criteria)
9. Digital low-pass filter	The dataset size employed for calculating the alienation/correlation factors functions as a digital low-pass filter, effectively suppressing ripples and attenuating certain harmonics	Some require additional digital low-pass filters. In cases of harmonic distortion, Fourier Transform filters harmonics, recommended for RMS calculations
10. CT saturation detection	The alienation/correlation concept can detect and evaluate CT saturation, enabling conventional digital relays to adjust the restraint factor/slope of their operating characteristics in order to increase the blocking zone during external faults with CT saturation extent	Most conventional methods require an additional CT saturation detector

Table 11 classifies the main approaches used for detecting and assessing unbalance and disturbances in three-phase voltage and current waveforms. Standardized indices are widely used in practice due to their simplicity and compliance with international standards; however, they often neglect phase-angle information or require relatively long data windows, which limits their sensitivity and speed. Signal-processing-based methods provide high-resolution disturbance detection and strong robustness against harmonics, but this comes at the expense of increased computational complexity, large data requirements, and sensitivity to parameter tuning, making them less suitable for fast protective relaying. Intelligent and analytical methods, including correlation- and alienation-based techniques, offer enhanced flexibility and sensitivity by exploiting signal relationships rather than absolute magnitudes. Nevertheless, some intelligent approaches require extensive training or complex modeling. The proposed alienation/correlation-based framework belongs to the analytical category and effectively overcomes these limitations by avoiding training requirements, reducing computational burden, and enabling fast, reliable unbalance and disturbance detection, which makes it particularly suitable for real-time protection and monitoring of synchronous machines.

**Table 11:** Categories of unbalance/disturbance detection and assessment methods for three-phase voltage and current waveforms.

Category	Typical methods / standards	Required inputs	Strengths	Limitations	Typical applications
1. Standardized indices	IEEE Std. 936 (Max–Min RMS)^43^, IEEE Std. 112 PVUR^44^, NEMA LVUR^45^, IEC/IEEE VUF^46,47^, MCD/ CMCD/ECD^48^	RMS voltages or sequence components	Simple, standardized, and widely adopted; LVUR enables fast detection; VUF has strong physical interpretation	LVUR neglects phase-angle deviations; VUF is computationally intensive and slower than half-cycle methods	Grid monitoring, standards compliance, basic relay settings
2. Signal-processing- based methods	Wavelet Transform (WT)^49^, Fractional Fourier Transform (FRFT)^50^, Fuzzy logic and cross- correlation^51^, PLL and symmetrical components^52^, Vsum & Vspace^53^	Time-domain voltage and current signals	High disturbance resolution; robust to harmonics; suitable for real-time power quality (PQ) disturbance classification	Requires large data windows; higher computational burden; sensitive to parameter tuning	Power quality analysis, online monitoring, PQ studies
3. Intelligent and analytical methods	Coherence^39–54^, Artificial Neural Networks (ANN)^14,55^, Alienation principle^56^, Coherence– Lagrangian methods^12^, Harmonic-based Decision Trees^10^	Voltages, currents, harmonics, extracted features	Flexible fault classification; capable of modeling nonlinear behavior; suitable for advanced diagnostics	ANN requires extensive training and may overfit; analytical models can be complex with limited interpretability	Synchronous machine fault diagnosis, advanced protection relays, predictive maintenance

## Implementation considerations

1. Implementation Feasibility: The proposed alienation-coefficient algorithm relies on simple time-domain correlation calculations (addition, multiplication, and averaging) over short data windows, resulting in significantly lower computational complexity than spectral- or AI-based schemes. Execution time tests with typical sampling rates and window lengths confirm that the computational load is well within the capabilities of standard relay-class microprocessors, enabling real-time operation without specialized hardware.
2. Detection Speed: The detection speed can be adjusted via the data window size, typically less than or equal to one periodic cycle. The window can be set to a full cycle or a fraction thereof to modify the total detection time for computing all 15 alienation indices. The microprocessor relay response depends on processor speed, program size, and software complexity.
3. Measurement Accuracy and Computational Efficiency: To minimize the effects of measurement errors on the alienation estimators, the following measures were adopted:

- Data window smoothing to reduce variance from short-term signal fluctuations, mitigating the impact of ripples, temporary faults, and DC offsets.
- Differential mode operation of the Data Acquisition Card (DAC).
- Proper grounding and bonding of the experimental setup.
- Use of anti-aliasing filters.
- Instrument transformers of identical type and accuracy class (both CTs and VTs).
- Use of monitoring and analysis tools, including meters, sensors, and software, to measure and record power quality parameters.

(4) Backup Protection Role: The proposed scheme functions as a backup relay for synchronous generator stator windings, coordinated with primary protection to ensure proper fault identification and clearance. For large-scale generators, the backup tripping time is typically set between 0.25 and 0.50 s, sufficient to compute all 15 indices while maintaining reliable protection.

## Conclusions

Synchronous generators are critical assets in the power grid, playing a pivotal role in ensuring system stability. Any malfunction in their protection systems can lead to severe disturbances and jeopardize overall grid reliability. To mitigate such risks and address potential failures of primary protection relays, it is essential to design secondary protection schemes with high operating speed and enhanced reliability.

This paper has presented a digital backup protection scheme contingent on the correlation-based alienation method and evaluates its performance under diverse fault disturbances and unbalanced operating conditions using a practical motor–generator set. The experimental setup, designed to emulate a three-phase power plant system, employs instrument transformers for voltage and current measurements, which are digitized via DAC and processed in LABVIEW for digital signal analysis and algorithm validation. This configuration has enabled comprehensive testing of the developed protection algorithm, allowing for real-time interaction between the system and the protection logic.

Experimental results demonstrate that the proposed scheme, based on alienation indices computed from three-phase voltage and current signals, provides comprehensive backup protection against synchronous generator faults. Building on the quantitative findings, the correlation-based alienation method demonstrates faster response, enhanced performance metrics, higher accuracy, and a more reliable assessment of unbalance and disturbance levels compared to the coherence-based alienation method applied to voltage and current signals. The results show that the protection’s security (SP) and dependability (DP) exceed 99.80%, while its reliability (RP) and accuracy (AP) surpass 99.60%. Furthermore, the proposed scheme achieves a malfunction rate of only 0.37%, compared to 1.48% in the earlier study that employed the coherence-based approach. In addition, an alienation-based tripping-time curve has been formulated for the backup protection scheme to ensure coordinated, time-delayed tripping under fault conditions, with its response time adjustable through a moving dataset to meet specific speed requirements.

## Supplementary Information

Below is the link to the electronic supplementary material.

Supplementary Material 1

## Data Availability

All data generated or analysed during this study are included in this published article [and its supplementary information files].
