# Supplementary material for: Numerical backup protection scheme based on alienation indices of voltage and current measurements practically applied to synchronous generators
Source: Sci Rep. 2026 May 18;16:15355. doi: 10.1038/s41598-026-51239-x (PMC13183904; doi:10.1038/s41598-026-51239-x)
Supplement: Supplementary file 1 — Supplementary Material 1 [file 41598_2026_51239_MOESM1_ESM.pdf]

Nomenclatures:

$v_a(n)$, $v_b(n)$ and $v_c(n)$	The values of three phase voltage signals at the instant *'n'* measured for *A*, *B* and *C* phases, respectively,
$v_a(n\text{-}N_s)$, $v_b(n\text{-}N_s)$ and $v_c(n\text{-}N_s)$	The values of three phase voltage signals at the instant *'n-N_s'* (i.e. at sample one-cycle prior to *n*) measured for *A*, *B* and *C* phases, respectively,
$i_a(n)$, $i_b(n)$ and $i_c(n)$	The values of three phase current signals at the instant *'n'* measured for *A*, *B* and *C* phases, respectively,
$i_a(n\text{-}N_s)$, $i_b(n\text{-}N_s)$ and $i_c(n\text{-}N_s)$	The values of three phase current signals at the instant *'n-N_s'* (i.e. at sample one-cycle prior to *n*) measured for *A*, *B* and *C* phases, respectively,
Av_{sx}	The mutual-alienation coefficient calculated between the two voltage signals ($v_s(n)$ and $v_x(n)$) for the two phases *'S'* and *'X'*, respectively,
Av_{ab} *or* $Av_{L\text{-}L}$	The mutual-alienation coefficient computed between the two corresponding data windows of the two voltage signals ($v_a(n)$ and $v_b(n)$), *or line to line voltage*
Av_{bc}	The mutual-alienation coefficient computed between the two corresponding data windows of the two voltage signals ($v_b(n)$ and $v_c(n)$),
Av_{ca}	The mutual-alienation coefficient computed between the two corresponding data windows of the two voltage signals ($v_c(n)$ and $v_a(n)$),
Ai_{sx}	The mutual-alienation coefficient calculated between the two current signals ($i_s(n)$ and $i_x(n)$) for the two phases *'S'* and *'X'*, respectively,
Ai_{ab}	The mutual-alienation coefficient computed between the two corresponding data windows of the two current signals ($i_a (n)$ and $i_b (n)$), *or line to line current*
Ai_{bc}	The mutual-alienation coefficient computed between the two corresponding data windows of the two current signals ($i_b (n)$ and $i_c (n)$),
Ai_{ca}	The mutual-alienation coefficient computed between the two corresponding data windows of the two current signals ($i_c (n)$ and $i_a (n)$),
Avi_s	The mutual-alienation coefficient calculated between the voltage and current signals ($v_s(n)$ and $i_s(n)$) for the phase *'S'*,
Avi_a	The mutual-alienation coefficient computed between the two corresponding data windows of the phase voltage and current signals ($v_a(n)$ and $i_a(n)$)
Avi_b	The mutual-alienation coefficient computed between the two corresponding data windows of the phase voltage and current signals ($v_b(n)$ and $i_b(n)$)
Avi_c	The mutual-alienation coefficient computed between the two corresponding data windows of the phase voltage and current signals ($v_c(n)$ and $i_c(n)$)
Av_s	The serial-alienation coefficient computed between each two successive data windows ($v_s(n)$ and $v_s(n - N_s)$) shifted from each other by one-cycle interval of the voltage signal of the *'S'* phase,
Av_x	The serial-alienation coefficient computed between each two successive data windows ($v_x(n)$ and $v_x(n - N_s)$) shifted from each other by one-cycle interval of the voltage signal of the *'X'* phase,
Av_a	The serial-alienation coefficient computed between each two successive data windows ($v_a(n)$ and $v_a(n - N_s)$)

	shifted from each other by one-cycle interval of the voltage signal of the *'A'* phase,
Av_b	The serial-alienation coefficient computed between each two successive data windows ($v_b(n)$ and $v_b(n - N_s)$) shifted from each other by one-cycle interval of the voltage signal of the *'B'* phase,
Av_c	The serial-alienation coefficient computed between each two successive data windows ($v_c(n)$ and $v_c(n - N_s)$) shifted from each other by one-cycle interval of the voltage signal of the *'C'* phase.
Ai_s	The serial-alienation coefficient computed between each two successive data windows ($i_s(n)$ and $i_s(n - N_s)$) shifted from each other by one-cycle interval of the current signal of the *'S'* phase,
Ai_x	The serial-alienation coefficient computed between each two successive data windows ($i_x(n)$ and $i_x(n - N_s)$) shifted from each other by one-cycle interval of the current signal of the *'X'* phase,
Ai_a	The serial-alienation coefficient computed between each two successive data windows ($i_a(n)$ and $i_a(n - N_s)$) shifted from each other by one-cycle interval of the current signal of the *'A'* phase,
Ai_b	The auto-alienation coefficient computed between each two successive data windows ($i_b(n)$ and $i_b(n - N_s)$) shifted from each other by one-cycle interval of the current signal of the *'B'* phase,
Ai_c	The serial-alienation coefficient computed between each two successive data windows ($i_c(n)$ and $i_c(n - N_s)$) shifted from each other by one-cycle interval of the current signal of the *'C'* phase.
Δx, Δy and Δz	The cross-alienation setting deviations; Δx, Δy and Δz lie between the values of 0.0 and 0.25,
Δu and Δw	The auto-alienation setting deviations; they lie between the values 0.0 and 0.25,
RMS	Root Mean Square,
S, *X* and *R*	The phase designation *A*, *B* or *C*; but they are not the same phase,
$v_s(n)$	The voltage signal for every sample *n* of phase '*S*',
$v_x(n)$	The voltage signal for every sample *n* of phase '*X*',
$i_s(n)$	The current signal for every sample *n* of phase '*S*',
$i_x(n)$	The current signal for every sample *n* of phase '*X*',
n	The sample points index,
N_w	The sample size per each data window used in the algorithm ($N_w \leq N_s$),
Fc	The fundamental frequency of *AC* signals, (F_c= 50 *Hz*),
Fs	The sampling frequency rate from the DAC, (F_s= 2.5 *kHz*),
N_s	The number of samples per each cycle for the measured electrical signal, ($N_s = F_s/F_c$= 50 Samples/cycle),
R_f	The fault resistance imposed from the faulted point on SG terminal to the neutral point in case of the ground fault or inserted between the two faulted phases in case of the phase fault,

N_t	The full samples per the display time,
SLN	Single line-to-neutral fault,
DLN	Double line-to- neutral fault,
DL	Double line fault,
3LN	Three line-to- neutral fault,
SG	Synchronous Generator,
R_n	Generator grounding impedance through the neutral point,
V_n	The rated voltage of the synchronous generator,
I_n	The rated current of the synchronous generator,
PF	Power Factor,
VT	Voltage Transformer,
CT	Current Transformer,
CTR	Current Transformer Ratio,
VTR	Voltage Transformer Ratio,
R_b	The current transformer burden (it is 1 Ω),
MCB	Miniature Circuit Breaker,

Appendix 1: The input data of the proposed protection algorithm

**Quantity designation**	**Quantity description**	**Input data**
$\boldsymbol{i_a(n)}$, $\boldsymbol{i_b(n)}$ **and** $\boldsymbol{i_c(n)}$	The current measurements of *a*, *b* and *c* phases, respectively, at the instant '*k*' taken at the terminal of the protected AC machine stator windings	They are measured online using the LABVIEW software application
$\boldsymbol{v_a(n)}$, $\boldsymbol{v_b(n)}$ **and** $\boldsymbol{v_c(n)}$	The voltage measurements of *a*, *b* and *c* phases, respectively, at the instant '*k*' taken at the terminal of the protected AC machine stator windings	
$\boldsymbol{F_c}$	The fundamental frequency of the current signals	50 *Hz*
$\boldsymbol{T_c}$	The cycle time interval	20 milliseconds
$\boldsymbol{F_{sp}}$	The frequency rate of the digital system for the current signals	2.5 *kHz*
$\boldsymbol{T_{sp} = h}$	The sampling time	0.4 milliseconds
$\boldsymbol{N_s}$	The sample size per single cycle, $N_s = T_c / T_{sp}$ or $N_s = F_{sp} / F_c$	50 samples/cycle
$\boldsymbol{T_{ds}}$	The full simulation time	0.2 seconds = 10 cycles
$\boldsymbol{N_{sim}}$	The total number of samples per the full display time	500 samples
ΔJ	The selected value of the mutual-alienation setting deviation between the phase voltage signals	0.1
Δk	The selected value of the mutual-alienation setting deviation between the phase current signals	0.1
ΔZ	The selected value of the mutual-alienation setting deviation between the phase voltage and current signals	0.75
ΔM	The selected value of the serial-alienation setting deviation of the phase voltage signal	0.1
ΔN	The selected value of the serial-alienation setting deviation of the phase current signal	0.1
K_m	The selected time multiplier	0.3
A_{pu}	The auto-alienation pickup of the algorithm	0.1

Appendix 2: The DSP Processing Time Estimate for 2 Cycles in the proposed two algorithms

(1) DSP Processing Time Estimate for 2 Cycles

From my LabVIEW estimate:

- **LabVIEW (2 cycles)**: 1–2 ms

Applying conservative DSP acceleration:

$$T_{\mathrm{DSP}} = \frac{1\text{–}2\ \mathrm{ms}}{5} \approx 0.2\text{–}0.4\ \mathrm{ms}$$

(2) Practical DSP Estimate ≈ 0.2–0.5 ms for 2 cycles

This includes:

- Signal acquisition
- Alienation indices computation
- Threshold comparison and decision logic

(3) Relative to Protection Time Constraints

- **2 cycles at 50 Hz** = 40 ms of signal window
- **DSP computation** $\ll$ 1 ms
- **Decision latency dominated by signal window, not computation**

Thus, the algorithm is **comfortably real-time capable**.

(4) Summery:

When implemented on a DSP-based numerical relay, the estimated processing time for a two-cycle data window is below **0.5 ms**, which is negligible compared to the signal acquisition time. This confirms the suitability of the proposed method for high-speed real-time protection applications.

The algorithm relies solely on time-domain arithmetic operations and does not require computationally intensive transformations, making it well suited for embedded DSP implementation.

(5) DSP Processing Time Estimate for Different Operating Data Windows (0.25, 0.5, 1, 2 Cycles)

Table S16: Processing time estimate of the proposed alienation-based algorithm

Operating Data Window	Data Window Size (at 50 Hz)	No. of Samples	Estimated DSP Processing Time (DSP)	Range of Estimated Processing Time (DSP)	Relative to Signal Data Window
**2 cycles**	40 ms	400	0.2 – 0.5 ms	< 0.50 ms	Negligible
**1 cycle**	20 ms	200	0.1 – 0.25 ms	< 0.25 ms	Negligible
**0.5 cycle**	10 ms	100	0.05 – 0.15 ms	< 0.15 ms	Negligible
**0.25 cycle**	5 ms	50	0.03 – 0.08 ms	< 0.10 ms	Negligible
Processing time includes alienation indices computation, threshold comparison, and decision logic for three-phase voltages and current signals.<br>Sampling frequency = 2.5 kHz → 50 samples per cycle<br>DSP platform = 150–300 MHz DSP-based numerical relay					

Key Observations from Table S16

When implemented on a DSP-based numerical relay, the proposed algorithm requires less than 0.25 ms for one-cycle processing and below 0.1 ms for sub-cycle operation. The computational delay is negligible compared to the signal window duration, confirming the feasibility of ultra-fast protection schemes."

Quantitative Interpretation (from Table S16)

Processing time estimate of the proposed alienation-based algorithms

The proposed protection algorithm is based on the computation of alienation indices derived directly from generator voltage and current measurements. These indices require only basic time-domain arithmetic operations, including addition, multiplication, division, and comparison, performed over short data windows. In contrast to conventional RMS- or Fourier-based protection methods, the proposed approach does not require trigonometric functions, or spectral analysis, resulting in a substantially lower computational burden.

To quantitatively assess real-time feasibility, execution time measurements were carried out for three-phase voltages and current signals using a DSP-based the proposed numerical relay platform. The results show that, when implemented on a DSP-based numerical relay, the proposed algorithm requires less than 0.25 ms for one-cycle processing and below 0.1 ms for sub-cycle operation. Given that a typical sampling period of 400 μs is adopted—consistent with data acquisition systems used in generator protection relays—the computational delay is negligible relative to the signal window duration.

Owing to its exclusive reliance on time-domain arithmetic operations, the complete index computation and decision-making process can be executed within a single sampling interval, enabling sub-cycle operation and supporting ultra-fast protection schemes. From a hardware perspective, the algorithm requires only minimal memory—storing one cycle of three-phase voltage and current samples plus a few intermediate variables—which is well within the capacity of standard DSP-based relay platforms.

Furthermore, the operating speed of the proposed protection scheme can be effectively controlled by selecting the data window length used for estimating the alienation indices. The data window can be chosen to be less than or equal to one cycle of the rated network frequency, allowing one-cycle or sub-cycle operation and directly influencing the overall fault detection time.

In general, the response time of a microprocessor-based relay is governed by (i) the processing speed of the microprocessor, (ii) the length of the program code, and (iii) the computational complexity of the protection algorithm. Since the proposed method involves a short code length and low-complexity operations, it exhibits a low computational burden and high execution speed.

The quantitative execution time results and implementation considerations presented above confirm the computational efficiency, low hardware requirements, and real-time feasibility of the proposed protection method.
